# Comprehensive analysis of the immunological implication and prognostic value of *CXCR4* in non-small cell lung cancer

**DOI:** 10.1007/s00262-022-03298-y

**Published:** 2022-10-29

**Authors:** Wei Guo, Qilin Huai, Bolun Zhou, Lei Guo, Li Sun, Xuemin Xue, Fengwei Tan, Qi Xue, Shugeng Gao, Jie He

**Affiliations:** 1 Department of Thoracic Surgery, National Cancer Center/National Clinical Research Center for Cancer/Cancer Hospital, Chinese Academy of Medical Sciences and Peking Union Medical College, Panjiayuannanli No 17, Chaoyang District, Beijing, 100021 China; 2 Key Laboratory of Minimally Invasive Therapy Research for Lung Cancer, Chinese Academy of Medical Sciences, Beijing, China; 3 Department of Pathology, National Cancer Center/National Clinical Research Center for Cancer/Cancer Hospital, Chinese Academy of Medical Sciences and Peking Union Medical College, Beijing, China

**Keywords:** CXCR4, Non-small cell lung cancer, Immune cells, Immunotherapy, Prognostic signature

## Abstract

CXCR4 (C-X-C chemokine receptor type 4) is the most commonly expressed of all chemokine receptors in malignant tumors. However, studies on CXCR4 in non-small cell lung cancer (NSCLC) tumor immune microenvironment, including those determining its immune efficacy and prognostic potential, are still scarce. Therefore, in this study, we determined the ability of CXCR4 to predict immunotherapy response and prognosis in NSCLC using immunohistochemical staining and RT-PCR, respectively, in two independent cohorts from the National Cancer Center of China. We analyzed transcriptome sequencing data and clinical information from multiple public databases to assess immune cell infiltration in NSCLC and constructed immune risk prognostic signatures based on CXCR4-related immunomodulators. We found that immune cell infiltration is significant differences in NSCLC tissues and is moderately correlated with CXCR4 expression. High CXCR4 expression was significantly associated with poor prognosis in NSCLC patients and a higher response rate to immunotherapy. The ROC curve showed that CXCR4 expression exhibited excellent performance in predicting the efficacy of immunotherapy in NSCLC. We identified 30 CXCR4-related immunomodulators in lung adenocarcinoma (LUAD) and lung squamous cell carcinoma (LUSC) and constructed immune prognostic signatures based on CXCR4-related immunomodulators and CXCR4-related mutant genes. The signature-based prognostic risk score showed good performance in predicting patient prognosis in both LUAD and LUSC; high risk scores were significantly associated with poor prognosis (*P* < 0.0001) and was established as an independent prognostic factor by multivariate Cox regression. We postulate that CXCR4 is a potential predictive marker of immunotherapy efficacy in NSCLC and should be used in clinical settings. Moreover, the constructed signatures may be valuable in predicting patient prognosis in NSCLC.

**Supplementary Information:**

The online version contains supplementary material available at 10.1007/s00262-022-03298-y.

## Introduction

According to 2020 GLOBOCAN statistics, there were approximately 2.2 million new cases of lung cancer; lung cancer has the highest mortality rate and the second-highest incidence rate among various cancers, accounting for approximately 18% of total cancer-related deaths. It is a highly malignant tumor [1]. According to histopathological type, about 85% of lung cancers are classified as non-small cell lung cancer (NSCLC), of which lung adenocarcinoma (LUAD) and lung squamous cell carcinoma (LUSC) are the most common subtypes [2]. Despite the progress in screening technology represented by low-dose computed tomography and multidisciplinary treatment in recent years, many patients are diagnosed at the middle and late stages owing to the large patient base and high heterogeneity of the disease; consequently, the 5-year survival rate of NSCLC patients is still less than 20% [3–5]. The emergence of molecular targeted therapy and immunotherapy has brought new treatment prospects for patients who miss early surgical treatment. NSCLC patients have benefitted variously after different immune checkpoint inhibitor treatments including nivolumab, pembrolizumab, atezolizumab, sintilimab, etc. [6–8]. These inhibitors enhance the cytotoxicity of immune effector cells by affecting cytotoxic T-lymphocyte antigen-4 (CTLA4), programmed cell death protein 1 (PD-1), and PD-1 ligand (PD-L1), and improving the tumor microenvironment (TME) to resist tumor cell growth [9]. However, as with conventional targeted therapy, only a proportion of NSCLC patients benefit from immunotherapy. Therefore, identifying valuable and reliable biomarkers for predicting patient prognosis and anti-PD-1 immunotherapy response is crucial as it can benefit the diagnosis and treatment of NSCLC patients.

There exist 48 chemokines belonging to four sub-families; they interact with seven-transmembrane G protein-coupled receptors (GPCRs) and affect the transport of immune cells under different conditions in the body [10]. In recent years, many studies have shown that chemokine/chemokine receptor loops interact in autocrine and paracrine forms to promote tumor cell survival and growth and accelerate tumor neo-angiogenesis [11, 12]. C-X-C chemokine receptor type 4 (CXCR4), also known as CD184, is the most commonly expressed chemokine receptor in malignant tumors [13, 14]. CXCR4 is a specific chemokine receptor of CXCL12, which is highly expressed on human progenitor cells and stem cells, including cancer stem cells. The CXCR4/CXCL12 axis has been extensively studied and shown to promote cancer cell migration, invasion, and metastasis in a variety of cancers, such as lung cancer, liver cancer, breast cancer, prostate cancer, and esophageal cancer [15–19]. CXCR4 inhibitors have been shown to significantly improve the TME in pancreatic cancer and increase the sensitivity of tumors to immune checkpoint inhibitors, increasing the benefits of immunotherapy in pancreatic cancer [20]. Therefore, CXCR4 may be a potential candidate immune-related prognostic marker. However, the potential of CXCR4 as a tumor immune prognostic marker has not been extensively studied, especially in NSCLC. To the best of our knowledge, the prognostic value of CXCR4 in NSCLC has been assessed in small-scale samples [21] but the relationship between CXCR4 and immune markers has not been established.

In this study, we analyzed the immune and mutational status of CXCR4 and determined the prognostic potential of CXCR4 in NSCLC in a large sample cohort from the National Cancer Center of China (NCC). We also explored the ability of CXCR4 to predict the efficacy of immunotherapy in NSCLC patients based on CXCR4 mRNA expression and corresponding anti-PD-1 immunotherapy response in another immunotherapy cohort. Finally, we identified immunomodulators closely related to CXCR4 in NSCLC and constructed immune prognostic signatures for LUAD and LUSC patients.

## Materials and methods

### Patients and tissue samples

As LUAD and LUSC are the most common NSCLC subtypes, this study assessed clinical and expression profile data of LUAD and LUSC patients. The experimental part of our study involves a retrospective study of NSCLC patients from the National Cancer Center/Cancer Hospital, Chinese Academy of Medical Sciences (NCC/CAMS, Beijing, China), including 242 LUAD patients and 188 LUSC patients (Cohort 1, Table 1), that underwent R0 surgical resection. All samples used in this study are stored in the NCC Biobank. Follow-up strategy: The follow-up period was from 2006 to 2014. Patients were required to review and record their survival in the outpatient clinic every 3–6 months for the first 2 years after the operation, and once a year thereafter. The final follow-up confirmation time was March 4, 2019. Patients that met the following inclusion criteria were enrolled in the study of cohort 1: (1) patients pathologically confirmed with LUAD or LUSC; (2) patients with no distant metastasis confirmed by computed tomography and not undergone radical surgery R0 resection. Patients with the following characteristics were excluded from the study: (1) patients undergone radiotherapy, chemotherapy, and immunotherapy before surgery; (2) patients whose clinical information was lost, (3) or postoperative follow-up data was lacking.

**Table 1:** Clinical characteristics of the NSCLC patients (NCC cohort 1)

Characteristics	LUAD group (*n* = 242)	LUSC group (*n* = 188)
Age, years		
< 60	122	87
> 60	120	101
Sex		
Male	144	181
Female	92	7
Smoking history		
Yes	109	174
No	133	13
Stage		
I	81	34
II	108	140
III	107	84
OS state		
Alive	82	78
Death	160	110

In addition, 13 LUAD patients and 33 LUSC patients (Cohort 2, Table 2), who received neoadjuvant therapy with immunotherapy combined with chemotherapy were included in this study. The operation was performed 3–4 weeks after drug withdrawal. The obtained tumor tissue was used for subsequent RT-PCR and immunohistochemistry. Patients who met the following key inclusion criteria were included in the study of cohort 2: (1) Patients aged between 18 and 70 years old with Eastern Cooperative Oncology Group (ECOG) status score of 0–1; (2) Patients with stage IIA–IIIB who were diagnosed with NSCLC by positron-emission tomography–computed tomography (PET–CT) and histopathology and judged to be resectable (stage IIIB patients are limited to T3N2); (3) Have not received systemic chemotherapy, radiotherapy, surgery or targeted therapy; (4) The EGFR driver gene was negative and the organ function was normal. The main exclusion criteria were immunodeficiency, ongoing systemic immunosuppressive therapy, active autoimmune or infectious disease, and inoperability due to a clinically unexpected accident. Assess patient response to immunotherapy according to Response Evaluation Criteria in Solid Tumors (RECIST) version 1.1 [22]. Briefly, all patients underwent comprehensive evaluation with CT or PET–CT before and after treatment. Before treatment, the lesion measurement requirement for each patient was the summation of the diameters of all target lesions (including the longest diameter of non-lymph node lesions and the short diameter of lymph node lesions) as a reference value for disease baseline. The efficacy evaluation after treatment is divided into the following three categories: CR (complete response, all target and non-target lesions disappear, all lymph nodes must be non-pathological < 10 mm); PR (partial response, at least 30% reduction in the sum of target lesion diameters compared with baseline); SD (stable disease, target lesion reduction does not achieve PR, enlargement does not achieve PD). Representative imaging pictures of each treatment response are shown in Supplementary Figure 1.

**Table 2:** Clinical characteristics of the NSCLC patients with neoadjuvant immunotherapy (NCC cohort 2)

Characteristics	LUAD group (*n* = 13)	LUSC group (*n* = 33)
Age, years		
< 60	6	17
> 60	7	16
Sex		
Male	8	29
Female	5	4
Smoking history		
Yes	7	26
No	6	7
Stage		
II	5	18
III	8	15
Response evaluation		
CR	0	2
PR	4	20
SD	9	11

This study was performed in accordance with the Declaration of Helsinki and was approved by the National Cancer Center/Cancer Hospital Ethics Committee. All patients provided written informed consent.

### Acquisition of NSCLC sample expression profile data

*CXCR4* mRNA expression profile data and mutation annotation data were downloaded from The Cancer Genome Atlas (TCGA) database. In addition, the expression profile of the LUAD sample data set (535 cancer tissues and 59 normal tissues) and the LUSC sample data expression profile (502 cancer tissues and 49 normal tissues) were obtained from TCGA. All RNA expression data were converted from readings per kilobase pairs/million map reads (RPKM) to transcripts per million (TPM). All expression profile data were used for subsequent tumor-infiltrating immune cell analysis, gene differential expression, and *CXCR4*-related mutation expression profile determination.

### The tumor-infiltrating immune cell landscape in NSCLC and its correlation with CXCR4

Cell Type Identification By Estimating Relative Subsets Of RNA Transcripts (CIBERSORT) is a deconvolution algorithm generated based on the level of gene expression, which can quantify and evaluate 22 types of immune cells in a complex gene expression profile [23, 24]. We analyzed the expression profiles of LUAD and LUSC patients from the Cancer Genome Atlas (TCGA) database based on the CIBERSORT algorithm, to explore their corresponding TMEs and their correlation with CXCR4 expression. An absolute value of R greater than 0.25 was considered relevant, and a *P* value < 0.05 was considered significant. In addition, Tumor Immune Estimation Resource (TIMER) is a database that integrates tumor immunology, clinical, and genomic characteristics, which can comprehensively analyze the immune infiltration of various cancer types [25]. We used TIMER to evaluate the impact of CXCR4 copy number alternations (CNAs) on the level of immune cell infiltration in the NSCLC tumor microenvironment. Using "Gene" and "Correlation" modules to explore the correlation between CXCR4 in NSCLC, immune cells, and related markers.

### Evaluation of CXCR4 expression for predicting immunotherapy response

The potential value of CXCR4 in predicting immunotherapy response in NSCLC has also been comprehensively explored. First, the expression data of classical immune checkpoints including PD1 (PDCD1), PDL1 (CD274), CTLA4, LAG3, GAL9 (LGALS9), TIM-3 (HAVCR2), TIGIT and PD-1LG2 (PDCD1LG2) were obtained from the TCGA database to analyze the differences and correlations between CXCR4 expression and these markers. More importantly, we added the following newly developed and widely recognized immune response markers: tumor mutation burden (TMB), immunophenoscore (IPS), neoantigen and Tumor Immune Dysfunction and Exclusion (TIDE) score, to further evaluate the potential immunotherapy response prediction performance of CXCR4 [26]. TMB, IPS score, and neoantigen data were obtained from The Cancer Immunome Atlas (TCIA, https://tcia.at/home). TIDE, an algorithm that simulates two types of tumor immune escape (T cell dysfunction and T cell exclusion), has been shown to outperform other known immunotherapy markers in predicting immunotherapy response [27]. The TIDE score, T cell dysfunction score, and T cell exclusion score were obtained based on CXCR4 expression and relevant data declared on the TIDE (http://tide.dfci.harvard.edu) website. The median expression of CXCR4 was the grouping criterion, and *P* values < 0.05 were considered significant.

### Gene set enrichment analysis

Gene set enrichment analysis (GSEA) is a powerful algorithm that can predict the signaling pathways involved with certain genes [28]. According to the median expression of CXCR4, samples were divided into high and low expression samples, and the pre-defined gene set was sorted. Hallmark serves as the predetermined gene set for this CXCR4 tissue level. Subsequently, we extracted RNA-seq data from 188 lung cancer cell lines from the Cancer Cell Line Encyclopedia (CCLE) database, which covers 1457 cell lines and 84,434 gene biological information [29]. Pathways with *P* < 0.05 and false discovery rate (FDR) < 0.25 were considered significant.

### Determination of immunomodulators and their relationship with CXCR4

TISIDB (http://cis.hku.hk/TISIDB/) is a website based on PubMed literature database mining, TME sequencing data, and multiple sets of information from TCGA. It can explore the interaction between a tumor and the immune system [30]. We determined immunostimulators and immunoinhibitors significantly related to CXCR4 from TISIDB. We fed these immunomodulators into the STRING database to generate a protein–protein interaction (PPI) network and explore their significance in NSCLC [31]. We also performed Gene Ontology (GO) annotation and Kyoto Encyclopedia of Genes and Genomes (KEGG) pathway enrichment analysis for identifying immunomodulators with significant correlation with CXCR4.

### CXCR4-related immunomodulator signature and nomogram construction

The immunostimulators and immunoinhibitors showing significant correlation with LUAD and LUSC were identified, and the Akaike information criterion in the Cox model was used to screen variables in order [32]; a prognostic signature was accordingly constructed. The risk score of each immunomodulatory was calculated as follows: risk score = gene (variable) expression × risk coefficient. After calculation and screening, the immune signature of LUAD and LUSC with genes with the strongest prognostic ability was obtained. Based on the median risk score, NSCLC patients were divided into high-risk groups and low-risk groups to assess NSCLC risk and overall survival (OS). The receiver operating characteristic (ROC) curve and the area under the ROC curve (AUC value) were used to evaluate the accuracy of predicting prognosis. Univariate and multivariate Cox regression analysis was performed on age and TNM staging to determine independent prognostic factors in NSCLC patients. To further determine the prognostic value of the signature, we constructed a nomogram to provide a predictive model for the disease progression of each NSCLC patient [33].

### Immunohistochemical (IHC) staining and evaluation of CXCR4 expression

A total of 242 LUAD tissues and 188 LUSC tissues from the NCC biological specimen library were fabricated into a tissue microarray (TMA) and subjected to IHC using the rabbit polyclonal CXCR4 (1:100, HPA002037; Sigma-Aldrich, St. Louis, MO) antibody. The results of CXCR4 antibody staining were independently reviewed and recorded by two experienced pathologists at our hospital. The level of CXCR4 expression in each tissue was determined by calculating the H-score, which is the sum of the product of the percentage of positive cells and corresponding staining intensity. The ordinal values for the percentage of positive cells are as follows: 0 (0–25% positive staining), 1 (25–50% positive staining), 2 (> 50% positive staining). The ordinal values for staining intensity are as follows: no staining, 0; weak staining, 1; moderate staining, 2; strong staining, 3. Samples with an H-score ≤ 1 were classified in the CXCR4 low expression group and those with an H-score ≥ 2 were classified in the high expression group. In addition, cohort 2 was subjected to IHC staining using the same technique to obtain information on the correlation between CXCR4 expression and immunotherapy response. Due to the fact that some patients obtained less pathological tissue after surgery and there were cases with CR, we finally only stained 26 samples, including 10 LUAD and 16 LUSC.

### RNA preparation and RT-PCR

Tumor tissues from an independent immunotherapy cohort of 13 LUAD patients and 33 LUSC patients in our hospital were collected ensuring that they contain more than 70% tumor cells. According to the standard RNA isolation protocol, Trizol (Invitrogen, Carlsbad, CA, USA) reagent was used to extract total RNA from tumor cells. Then, 1 µg total RNA was used to synthesize complementary DNA (cDNA) for RT-PCR analysis. The expression of CXCR4 was calculated and quantified using the 2^−ΔΔCT^ method. Each group included three repeated wells. To visualize CXCR4 expression in each immunotherapy sample, quantified CXCR4 expression data were log2 transformed. The CXCR4 primers used in the RT-PCR analysis are as follows: CXCR4 forward: ACTACACCGAGGAAATGGGCT and CXCR4 reverse: CCCACAATGCCAGTTAAGAAGA. Post-treatment response assessed according to RECIST V1.1 criteria can be divided into CR (complete response), PR (partial response) and SD (stable disease). The response status of each sample and original data based on 2^−ΔΔCT^ CXCR4 expression levels are shown in Supplementary Table 1.

### Statistical analysis

The SPSS 23.0 software (New York, USA) was used to statistically analyze clinical data from the NCC cohort. The expression of CXCR4 and clinicopathological parameters were analyzed using the chi square test and Fisher's exact test. Kaplan–Meier survival analysis and log-rank test were used to analyze the influence of CXCR4 expression on patients’ survival and draw the survival curve. Univariate and multivariate Cox regression analyses were used to determine the independent prognostic factors in NSCLC. The R software (version 3.6.3) was used for bioinformatics analysis of data obtained from TCGA and other databases. The correlation between *CXCR4* expression and immune cell infiltration was analyzed using the Person correlation coefficient. An absolute value of *r* ≥ 0.25 was considered a strong correlation, and a *P* value < 0.05 is considered to be significant.

## Results

### The landscape of infiltrating immune cells in NSCLC

**Fig. 1:**
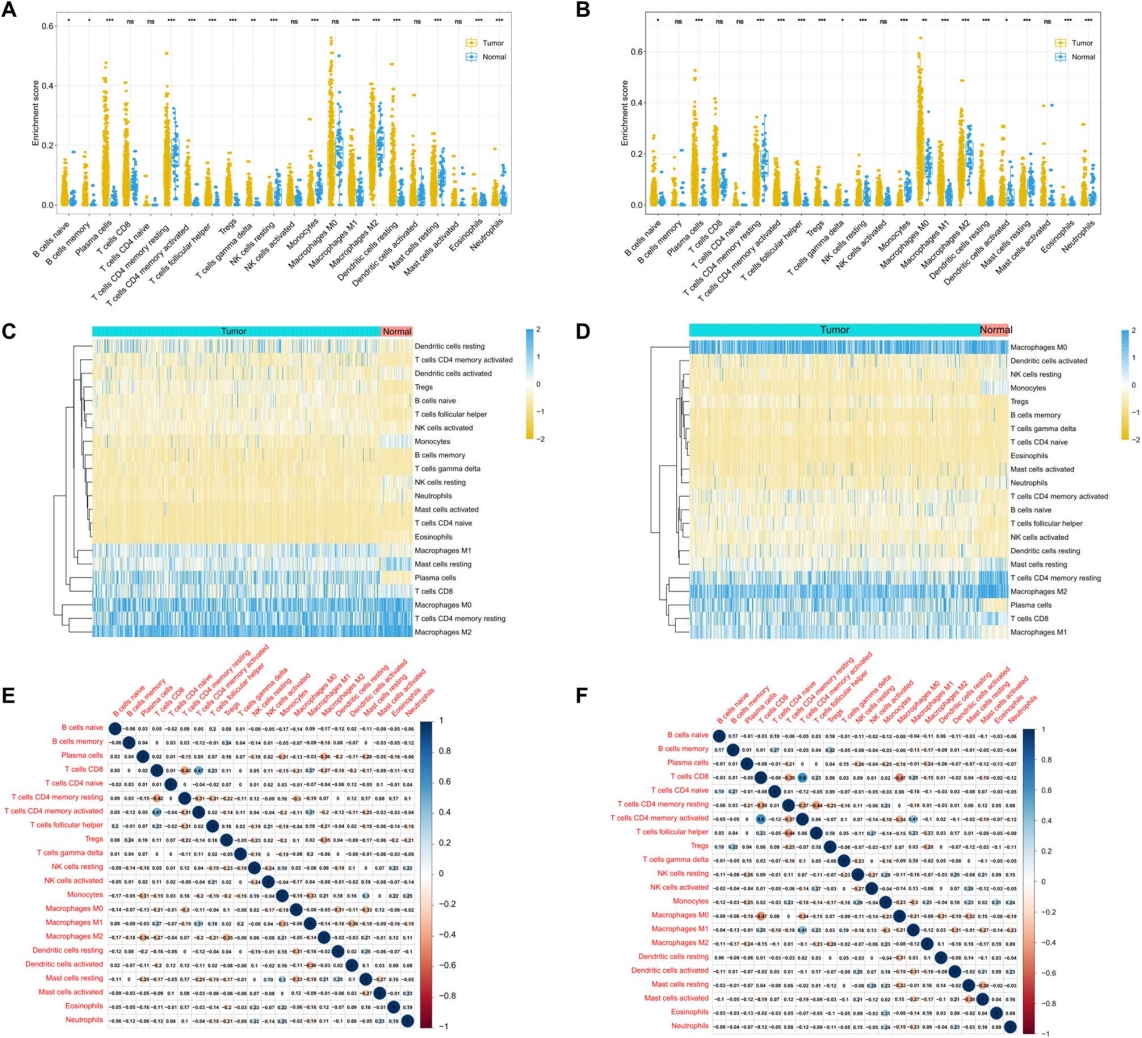
Evaluation of the proportions of 22 tumor-infiltrating immune cells in The Cancer Genome Atlas non-small cell lung cancer cohort. The dot plot and heatmap show the difference in the level of immune cell infiltration between tumor (yellow) and normal (blue) tissues in LUAD (**A**, **C**) and LUSC (**B**, **D**). The correlation heatmaps show weak to moderate correlations between different immune cell subgroups in LUAD **(E)** and LUSC **(F)** tissue samples

The CIBERSORT program was used to calculate the gene expression profiles of LUAD and LUSC patients from TCGA database, and the infiltration ratio of 22 immune cells was obtained. The infiltration levels of naïve B cells, plasma cells, T follicular helper cells, T regulatory cells, gamma delta T cells, M1macrophages, resting dendritic cells (DC), and eosinophils in tumor tissues were significantly higher in the tissues of LUAD and LUSC patients than in normal tissues. The infiltration levels of resting CD4 memory T cells, activated CD4 memory T cells, monocytes, M2 macrophages, resting mast cells, and neutrophils were significantly higher in tumor tissues than in normal tissues. Memory B cells were particularly significantly enriched in LUAD tissues, and M0 macrophages, and activated DC were significantly enriched in LUSC patients. The infiltration levels of resting natural killer (NK) cells were contrasting in LUAD and LUSC (*P* < 0.05, Fig. 1A, B). The differential infiltration level of 22 immune cells in LUAD and LUSC tumors and normal tissues were visualized in the form of heatmaps (Fig. 1C, D). In addition, the correlation heat map showed that 22 immune cells showed weak to moderate correlation in LUAD and LUSC tumors (Fig. 1E, F).

### Characteristics of mutant genes associated with *CXCR4* expression in LUAD and LUSC

To explore the potential mechanism by which *CXCR4* expression orchestrates changes in LUAD and LUSC, we identified mutant genes related to CXCR4. After dividing LUAD patients (Supplementary Figure 2A, C) and LUSC patients (Supplementary Figure 2B, D) into the high and low expression groups based on the median expression of *CXCR4*, we constructed a heatmap to determine the mutation frequency of mutant genes related to CXCR4 expression. Subsequently, based on the chi-square test, we found that the mutation frequency of *DNAH8*, *PAPPA2*, *SPHKAP*, *XIRP2*, and *ZNF804B* (Supplementary Figure 2E) was significantly different in the CXCR4 high expression and low expression groups of the LUAD cohort. Similarly, the mutation frequency of CSMD2, PCDH15, RELN, SI, and ZNF804A was significantly different in the CXCR4 high expression and low expression groups of the LUSC cohort (Supplementary Figure 2F).

### Correlation between CXCR4 and immune cell infiltration

To elucidate the underlying mechanism by which CXCR4 modulates the infiltration of different immune cells, we first investigated the correlation between major immune cells and CXCR4 expression. We found that, in LUAD, CXCR4 was moderately negatively correlated with tumor purity, and moderately positively correlated with the tumor infiltration of B cells, CD8+ T cells, CD4+ T cells, macrophages, neutrophils, and DC cells (Supplementary Figure 3A). The correlation between CXCR4 expression and major immune cells in LUSC was similar to that observed in LUAD but more significant, especially the correlation coefficient between CXCR4 and DC cells was 0.624 (Supplementary Figure 3B). Next, we analyzed the effect of somatic CNAs in *CXCR4* on immune cell infiltration. In LUAD, CNAs in *CXCR4*, including arm-level deletion and arm-level gain, significantly affected the infiltration levels of B cells, CD4+ T cells, macrophages, neutrophils, and DCs (Supplementary Figure 3C). In LUSC, arm-level gain and high amplication significantly affected the infiltration levels of B cells, CD4+ T cells, macrophages, neutrophils, and DCs (Supplementary Figure 3D). In addition, the correlation between CXCR4 expression and 22 immune cells in LUAD and LUSC was also assessed. Regulatory T cells (*r* = 0.26), B plasma cells (*r* = 0.3), M1 macrophages (*r* = 0.37), memory B cells (*r* = 0.46), resting CD4+ memory T cells (*r* = 0.52), gamma delta T cells (*r* = 0.26), CD8+ T cells (*r* = 0.5), T follicular helper cells (*r* = 0.4), activated NK cells (*r* = 0.34), and M2 macrophages (*r* = 0.46) showed a strong positive correlation with CXCR4 expression in LUAD (Supplementary Figure 4A). Immune cells, including naïve B cells (*r* = 0.3), regulatory T cells (*r* = 0.42), monocytes (*r* = 0.26), B cell plasma (*r* = 0.35), M1 macrophages (*r* = 0.49), memory B cells (*r* = 0.39), resting CD4+ memory T cell (*r* = 0.54), macrophages M0 (*r* = 0.25), CD8+ T cells (*r* = 0.56), follicular helper T cells (*r* = 0.43), activated NK cell (*r* = 0.31), and M2 macrophages (*r* = 0.55), showed a significant correlation with CXCR4 in LUSC (Supplementary Figure 4B).

### Potential value of CXCR4 in predicting immunotherapy response in NSCLC patients

To explore the possibility of CXCR4 predicting response to immunotherapy in NSCLC patients, we included PD1 (PDCD1), PDL1 (CD274), CTLA4, LAG3, GAL9 (LGALS9), TIM-3 (HAVCR2), TIGIT and PD-1LG2 (PDCD1LG2) as immune checkpoint-related candidate genes to evaluate their relationship with CXCR4. The results showed that CXCR4 was significantly positively correlated with the expression of these classical immune checkpoints in LUAD, and the correlation coefficients of PD1, CTLA4, TIM3, TIGIT and PD-1LG2 were greater than 0.5 (Supplementary Figure 5A). In addition, the above immune checkpoints were significantly up-regulated in the CXCR4 high expression group (*P* < 0.001, Supplementary Figure 5B). In LUSC, the differences and correlations between CXCR4 and immune checkpoints showed the same trend as LUAD (Supplementary Figure 6A, B). Newly developed and widely recognized immune response markers in recent years including TMB, IPS, neoantigen, TIDE score, T cell dysfunction score and T cell exclusion score are also used for comprehensive evaluation. In LUAD, we did not find significant differences in TMB, IPS and neoantigen levels between high and low CXCR4 expression groups, but found significantly lower TIDE scores, higher T cell dysfunction scores, and lower T cell exclusion scores in the CXCR4 high expression group, indicating that the CXCR4 high expression group may be more sensitive to immunotherapy (Supplementary Figure 7A). In LUSC, TMB, IPS, and T cell dysfunction scores were significantly increased in the CXCR4-high expression group, while TIDE scores and T cell exclusion scores were significantly decreased, also showing the potential of CXCR4 in predicting immunotherapy response (Supplementary Figure 7B).

**Fig. 2:**
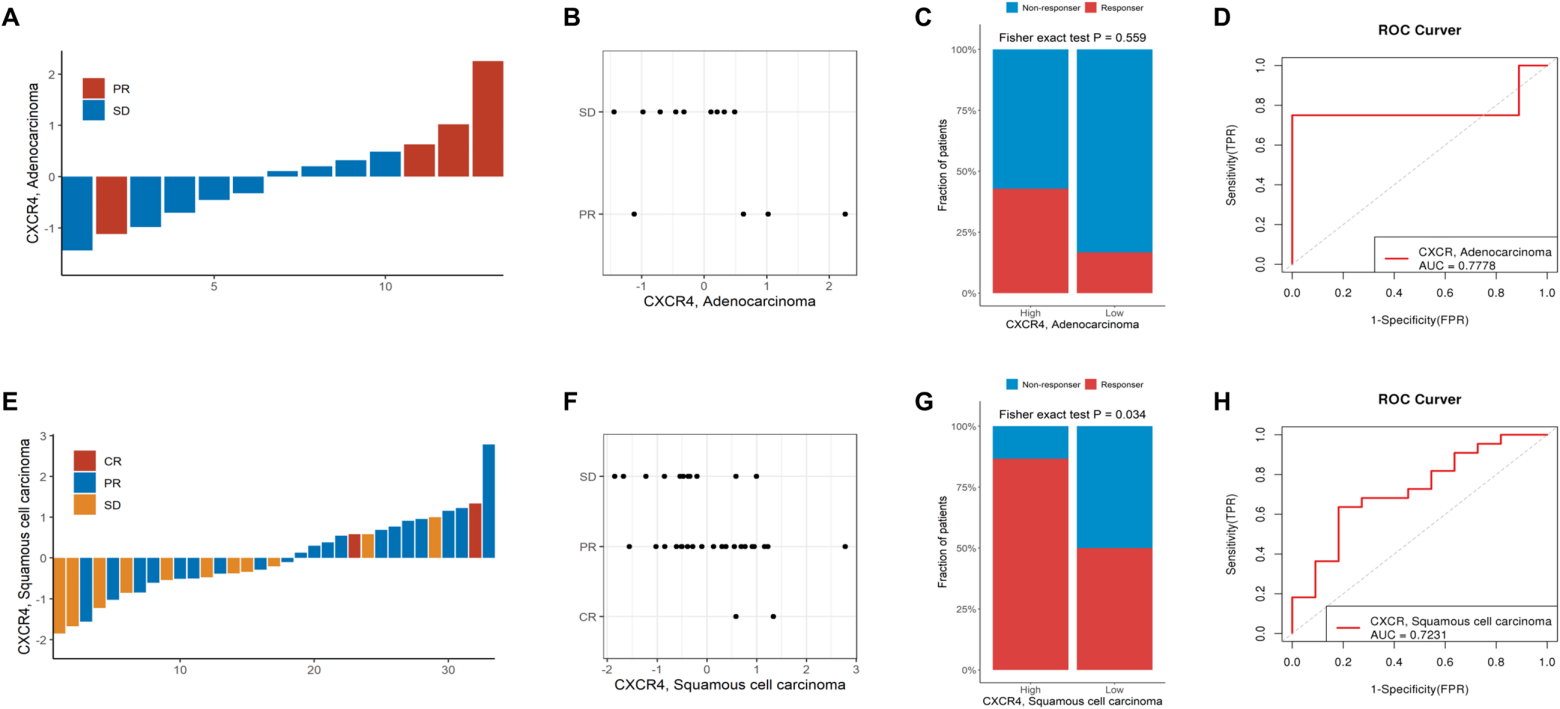
CXCR4 predicts the anti-PD-1 immunotherapy response in NSCLC. **A** and **B** Histogram and scatterplot of CXCR4 mRNA expression in LUAD patients treated with anti-PD-1 immunotherapy. **C** Histogram of the percentages of immunotherapy response rates and CXCR4 mRNA expression levels in LUAD. **D** ROC curve of CXCR4 in predicting the response to immunotherapy in LUAD. **E** and **F** Histogram and scatterplot of CXCR4 mRNA expression in LUSC patients treated with anti-PD-1 immunotherapy. **G** Histogram of the percentages of immunotherapy response rates and CXCR4 mRNA expression levels in LUAD. **H** ROC curve of CXCR4 in predicting the response to immunotherapy in LUSC. *CR* complete response, *PR* partial response, *SD* stable disease

Next, RT-PCR analysis was performed on patients (cohort 2) to verify the correlation between *CXCR4* expression and immunotherapy response. In LUAD patients, high *CXCR4* expression was largely consistent with better immunotherapy response. Three of the four patients with partial response (PR) had higher CXCR4 expression than the stable disease (SD) patients (Fig. 2A, B). The percentage histogram also showed that in LUAD, the response rate was higher in the CXCR4 high expression group than in the low expression group, but no significant p-value was obtained due to sample size limitation (Fig. 2C). Among LUSC patients, PR and complete response (CR) patients showed high CXCR4 expression (Fig. 2E, F). The percentage histogram showed that in LUSC, the response rate was higher in the CXCR4 high expression than in the low expression group (*P* < 0.05, Fig. 2G). The ROC curve showed that the AUC value of CXCR4 expression in the LUAD immunotherapy response cohort was 0.7778 and that in the LUSC immunotherapy response cohort was 0.7231, indicating the good potential of CXCR4 expression levels to predict immune efficacy (Fig. 2D, H). In addition, IHC staining was performed on cohort 2 samples to explore the correlation of CXCR4 expression with immunotherapy response. In the final 26 IHC staining samples, we analyzed in the LUAD, LUSC and overall three groups respectively, and found that the immunotherapy response between the high and low expression groups of CXCR4 did not reach statistical significance (Supplementary Figure 8A–C). However, among the 26 NSCLC patients, 38% of the samples had consistent CXCR4 expression at the RNA and protein levels, which requires further verification by expanding the samples (Supplementary Figure 8D). Representative IHC staining pictures are shown in Supplementary Figure 9.

### CXCR4 related biological processes and pathways

In addition, the signal pathways associated with the *CXCR4* upregulation in LUAD and LUSC were determined via GSEA. MYC targets, allograft rejection, DNA repair, and inflammatory response were the top four pathways enriched in LUAD (Supplementary Figure 10A). Notch signaling, TGF-beta signaling, apical junction, and epithelial–mesenchymal transition were the top four pathways enriched in LUSC (Supplementary Figure 10B). The top 10 Hallmark pathways of LUAD and LUSC are shown in Supplementary Table 2 and Supplementary Table 3, respectively. RNA-seq data of 118 lung cancer cell lines from the CCLE database revealed that glycosaminoglycan degradation, long-term depression, phosphatidylinositol signaling system, and galactose metabolism were the most enriched signaling pathways in lung cancer cells (Supplementary Figure 10C–F). The top 10 pathways in lung cancer, as determined by GSEA, are listed in Supplementary Table 4 in detail.

### Prognostic potential of *CXCR4* expression in NSCLC

**Fig. 3:**
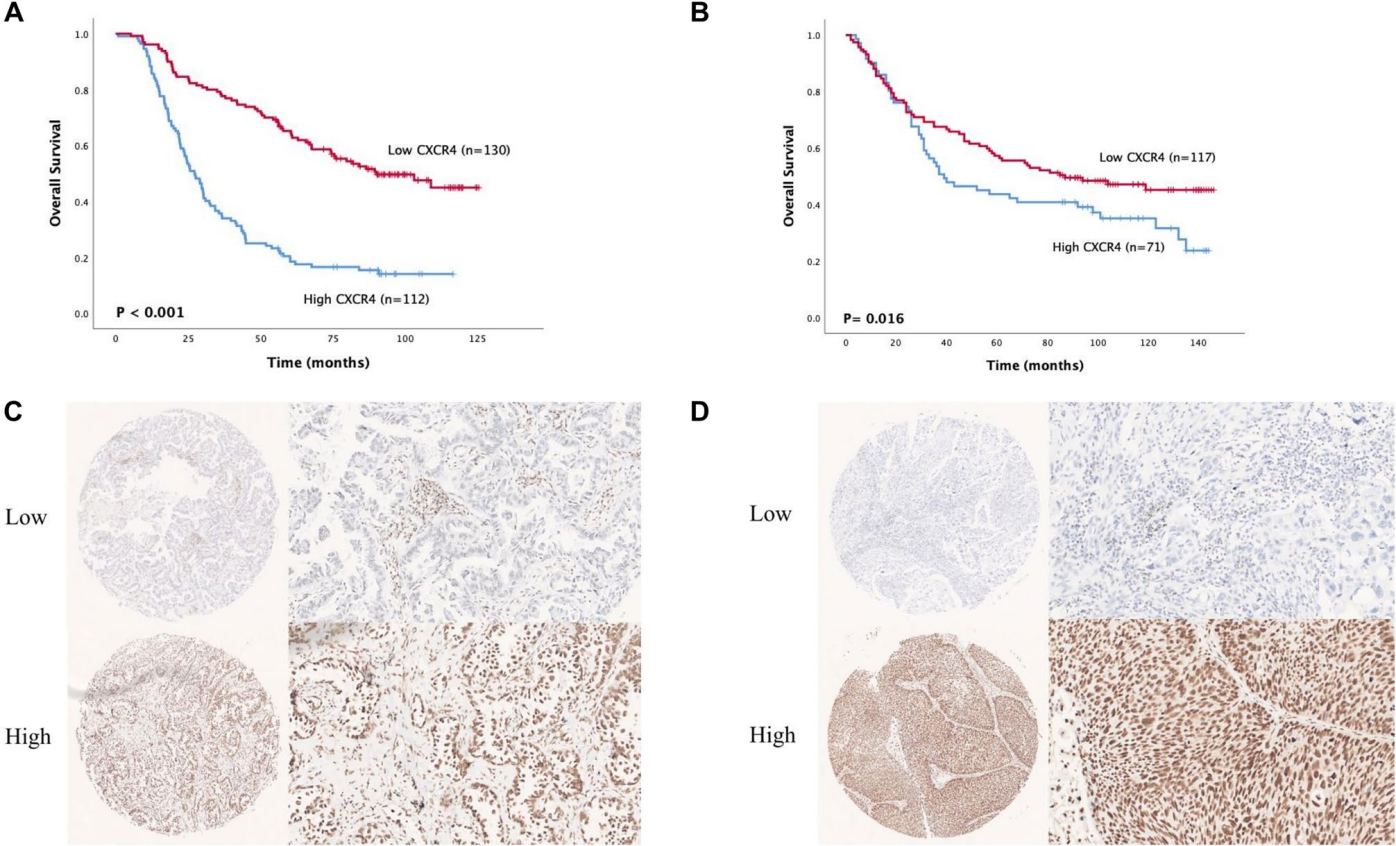
Survival analysis of CXCR4 in NSCLC based on the NCC cohort. Kaplan‒Meier survival analysis was performed on the relationship between CXCR4 and overall survival (OS) using NCC cohort LUAD (**A**) and LUSC (**B**) data. Representative immunohistochemical staining of CXCR4 protein in LUAD (**C**) and LUSC (**D**) tissue microarrays

To elucidate the prognostic value of CXCR4 expression in LUAD and LUSC, we performed IHC staining and survival analysis on independent samples from the NCC cohort and found that the high expression of CXCR4 was significantly related to the poor prognosis of LUAD (P < 0.001, Fig. 3A) as well as LUSC patients (*P* = 0.016, Fig. 3B). IHC staining of LUAD and LUSC tumor tissues with CXCR4 antibody is shown in Fig. 3C and D, respectively.

Further, univariate analysis of LUAD patients revealed that OS was significantly correlated with age, tumor length, differentiation, T stage, lymph node metastasis, TNM stage, and CXCR4 expression (*P* < 0.05). Multivariate analysis revealed that age, tumor length, differentiation, lymph node metastasis, and CXCR4 expression are independent prognostic factors in LUAD (*P* < 0.05, Supplementary Table 5). In LUSC patients, OS was significantly correlated with tumor length, differentiation, T stage, lymph node metastasis, and TNM staging (*P* < 0.05), and *t* differentiation, T staging, and TNM staging were identified as independent prognostic factors in LUSC (*P* < 0.05, Supplementary Table 6).

### Identification of CXCR4-related immunomodulators in NSCLC

**Fig. 4:**
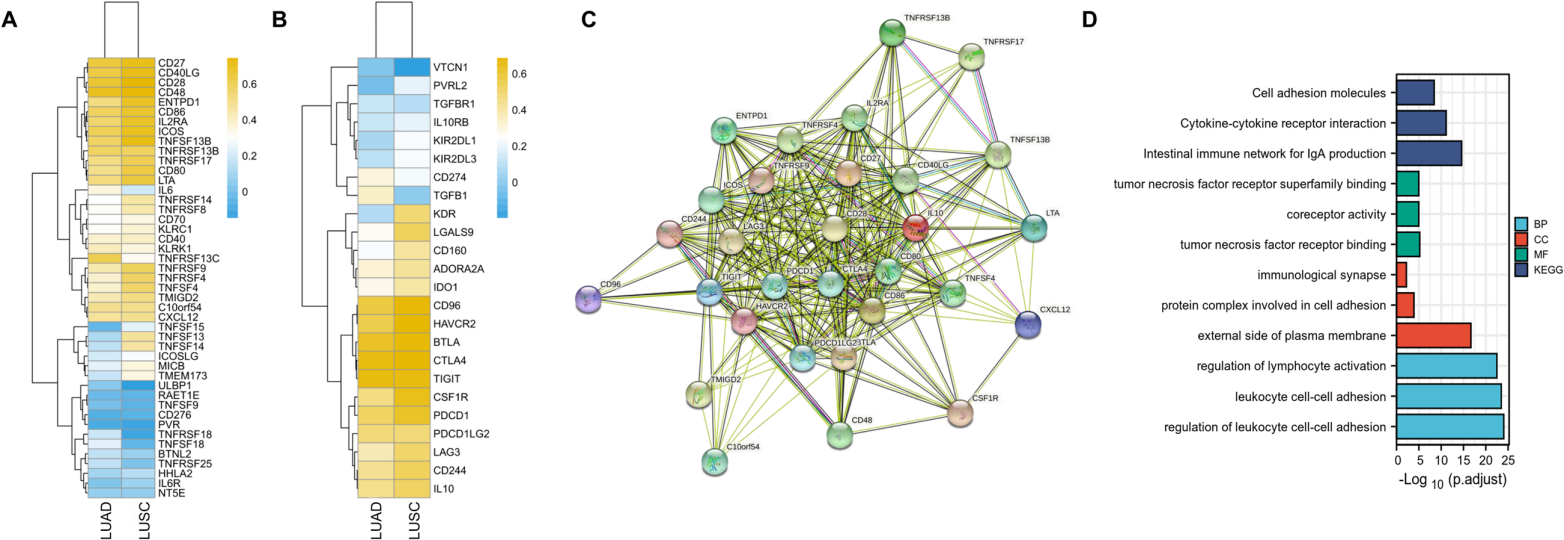
Identification of immunomodulators significantly related to CXCR4. Correlation heatmap of immunostimulators (**A**) and immunoinhibitors (**B**) related to CXCR4 in LUAD and LUSC. **C** Generated protein‒protein interaction network of 30 immunomodulators related to CXCR4 based on the STRING database. **D** GO and KEGG enrichment analyses of the biological functions and molecular pathways involved in the 30 CXCR4-related immunomodulators

To further elucidate the immune properties of CXCR4, we identified 19 immunostimulators (CD27, CD40LG, CD28, CD48, ENTPD1, CD86, IL2RA, ICOS, TNFSF13B, TNFRSF13B, TNFRSF17, CD80, LTA, TNFRSF9, TNFRSF4, TMIGD2, C10orf54, CXCL12) and 11 immunoinhibitors (CD96, HAVCR2, BTLA, CTLA4, TIGIT, CSF1R, PDCD1, PDCD1LG2, LAG3, CD244, IL10) that were significantly related to CXCR4 in LUAD as well as LUSC (Fig. 4A, B). Subsequently, we constructed a PPI network containing 30 significantly related immunomodulators, containing 30 nodes and 262 edges (Fig. 4C), in the STRING database. GO enrichment analysis revealed that the biological processes (BP), cell components (CC), and molecular functions (MF) involved in these 30 immunomodulators covered the regulation of lymphocyte activation, leukocyte cell–cell adhesion, external side of the plasma membrane, tumor necrosis factor receptor binding, etc. KEGG analysis revealed the top signal pathways immunomodulators participate in, including intestinal immune network for IgA production, cytokine-cytokine receptor interaction, and cell adhesion molecules (Fig. 4D).

### Construction of prognostic signatures based on CXCR4-related Immunomodulators

**Fig. 5:**
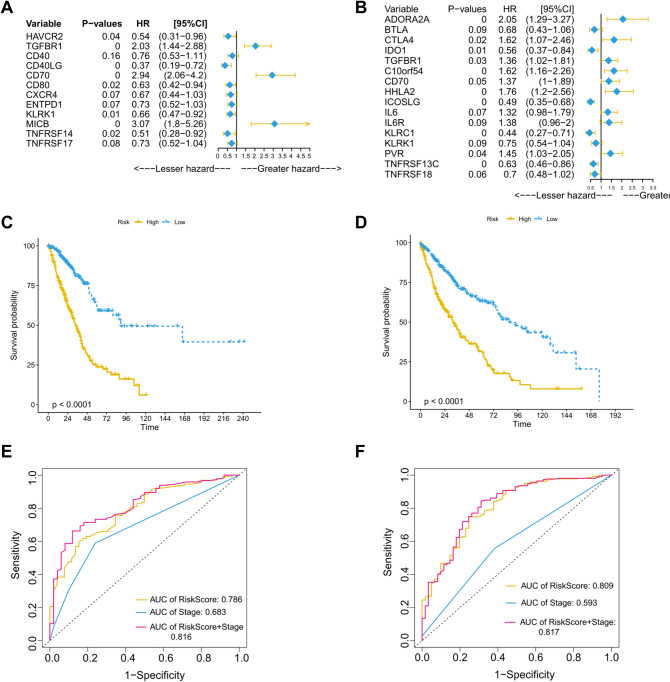
Construction of prognostic signatures based on 30 CXCR4-related immunomodulators in NSCLC. Multivariate forest plots in LUAD (**A**) and LUSC (**B**) identify immunomodulators with prognostic value. The Kaplan‒Meier curve shows that the risk score based on the prognostic signature is significantly related to the OS of LUAD (**C**) and LUSC (**D**) patients. The time-dependent ROC curve was used to evaluate the prediction efficiency of the prognostic signature in LUAD (**E**) and LUSC (**F**)

To explore the prognostic value of CXCR4, we constructed prognostic signatures based on CXCR4-related immunomodulators in LUAD and LUSC. In LUAD, we performed univariate and multivariate Cox regression analysis of the identified immunomodulators and found that HAVCR2, TGFBR1, CD40LG, CD70, CD80, KLRK1, MICB, and TNFRSF17 were significantly related to the OS of LUAD patients (*P* < 0.05). Subsequently, we adjusted the cut-off value to construct a prognostic signature. According to the minimum standard and the risk score of each immunomodulator, a signature containing 12 immunomodulators (HAVCR2, TGFBR1, CD40, CD40LG, CD70, CD80, CXCR4, ENTPD1, KLRK1, MICB, TNFRSF14, and TNFRSF17) was generated (Fig. 5A). The LUAD risk score was calculated as follows: risk score = (0.5448 * HAVCR expression) + (2.0341 * TGFBR1 expression) + (0.7648 * CD40 expression) + (0.3699 * CD40LG expression) + (2.9431 * CD70 expression) + (0.6282 * CD80 expression) + (0.6749 * CXCR4 expression) + (0.7319 * ENTPD1 expression) + (0.6553 * KLRK1 expression) + (3.0730 * MICB expression) + (0.5057 * TNFRSF14 expression) + (0.7327 * TNFRSF17 expression), and patients were divided into the high-risk and low-risk groups according to their median risk score. Kaplan–Meier survival analysis revealed that the OS of the high-risk group was significantly shorter than that of the low-risk group (*P* < 0.0001, Fig. 5C). The risk score and TNM staging-based area under the curve (AUC) of the high- ad low-risk groups were 0.786 and 0.683, respectively. The AUC calculated by combining the risk score and TNM staging was 0.816, indicating the good predictive performance of the signature (Fig. 5E). Similarly, in LUSC, we constructed a prognostic signature of 16 immunomodulators (ADORA2A, BTLA, CTLA4, IDO1, TGFBR1, C10orf54, CD70, HHLA2, ICOSLG, IL6, IL6R, KLRC1, KLRK1, PVR, TNFRSF13C, and TNFRSF18) based on CXCR4-related immunostimulators and immunoinhibitors (Fig. 5B). High risk scores were significantly related to the poor prognosis of LUSC patients (*P* < 0.0001, Fig. 5D). The ROC-AUC of the risk score was 0.809, and AUC calculated by combining the risk score and TNM staging was 0.817 (Fig. 5F).

**Fig. 6:**
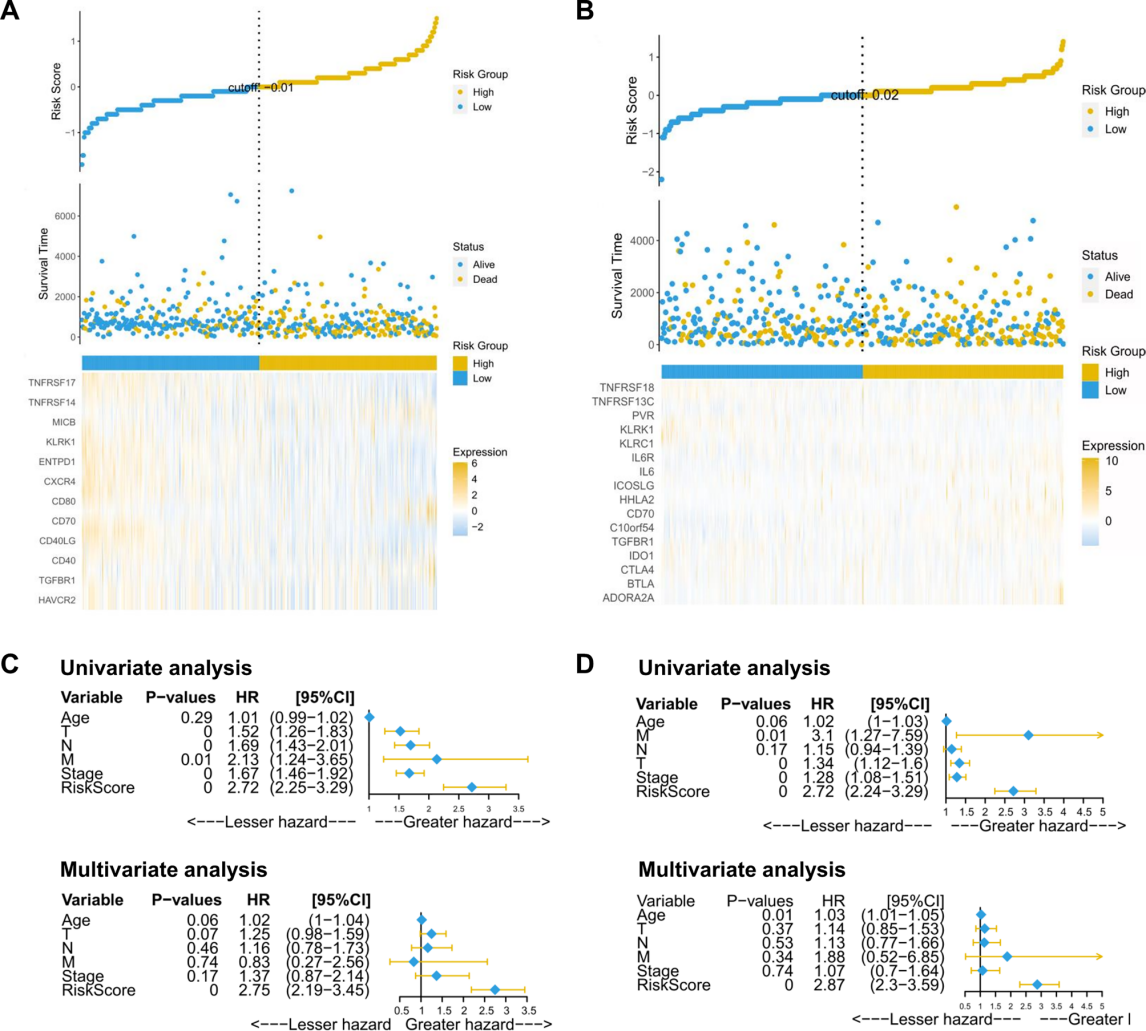
Evaluation of the prognostic value of the risk score. Distribution and gene expression profile of the risk score and patient survival status in LUAD (**A**) and LUSC (**B**). Univariate and multivariate Cox regression analyses of the clinical characteristics and risk scores of LUAD (**C**) and LUSC (**D**) patients

Figure 6A and B shows the risk score, OS, and survival status of each LUAD and LUSC patient through a dotted distribution map and a heatmap. The univariate Cox regression model showed that TNM pathological staging, and risk score were significantly correlated with OS in LUAD and LUSC, and multivariate Cox regression analysis confirmed that the risk score is an independent prognostic factor of LUAD (Fig. 6C, D). Finally, we constructed a prognostic nomogram based on the risk scores of LUAD and LUSC patients, which can calculate 3- and 5-year survival probabilities through different variables. The calibration curve shows that the model closely matches the ideal reference line (dotted line), corroborating the signature’s strong predictive ability (Supplementary Fig. 11A, B).

## Discussion

Lung cancer cells can affect the TME by expressing chemokine receptors, majorly CXCR4, and producing chemokines that regulate the transportation of immune and cancer cells [34]. Therefore, we studied the relationship between CXCR4 and tumor immunity features to determine the prognostic value of CXCR4 in NSCLC. As the characteristics of the immune microenvironment and prognosis are different for different histological subtypes of NSCLC [35], we separately studied LUAD and LUSC.

Previous studies have shown that high levels of tumor-infiltrating lymphocytes are significantly related to improved patient survival. Increased CD8+ T cells in tumors after immune checkpoint inhibitor treatment indicates a better clinical response [36, 37]. We found a variety of immune infiltrating cells in LUAD and LUSC. The infiltration of B cells was high in only LUAD, which is consistent with previous reports suggesting that adaptive immune responses are more dominant in LUAD [38]. In LUSC, only the population of M0 macrophages and activated DCs was significantly different between tumors and normal tissues. The increase in M0 macrophages has been shown to be related to the poor prognosis of patients with LUAD in the early clinical stage [39] but needs to be verified in LUSC. We also identified the CXCR4-related mutational spectrum. In LUAD, mutations in DNAH8, PAPPA2, SPHKAP, XIRP2, ZNF804B, and in LUSC, CSMD2, PCDH15, RELN, SI, and ZNF804A were significantly associated with CXCR4 expression. After searching the database, no reports of the relationship between CXCR4 and the above-mentioned significantly mutated genes have been found in tumors. These genes can further be probed to unveil the mechanism by which CXCR4 manifests its effects in LUAD and LUSC.

We also found that somatic CNAs in CXCR4 were associated with varying levels of immune cell infiltration in LUAD and LUSC. CXCR4 expression was found to be positively correlated with a variety of immune infiltrating cells in LUAD and LUSC, including regulatory T cells, resting CD4+ memory T cells, CD8+ T cells, follicular helper T cells, and activated NK cells, among others. Particularly, naïve B cells, monocytes, and M0 macrophages showed a significant correlation with CXCR4 expression in the LUSC cohort. In pancreatic ductal adenocarcinoma, CXCR4 blockers can reactivate CD8+ T cells in the TME, enhancing the benefits of PD-1 immunotherapy [40]. The CXCL12/CXCR4 pathway can recruit macrophages in oral squamous cell carcinoma through cancer-associated fibroblast (CAF) to promote the transformation of cancer stem cells [41].

In this study, CXCR4 showed a significant positive correlation with classical immune checkpoints including PD1, PDL1, CTLA4, LAG3, GAL9, TIM-3, TIGIT, PD-1LG2 in both LUAD and LUSC, and the expression of these markers was significantly increased in the CXCR4 high expression group. It should be noted that high expression of these immune checkpoints may lead to suppressed antitumor immunity in patients with high CXCR4 expression, and these findings also prompted our study of the prognostic value of CXCR4 in NSCLC patients. However, this phenomenon can also indicate that immunotherapy can alleviate the inhibitory effect of immune cells in the tumor microenvironment, and the effect of immunotherapy may be stronger [42]. In addition, we observed that the high expression of CXCR4 was accompanied by varying degrees of TMB, and IPS scores in LUAD and LUSC patients, and should be studied further in immune-independent cohorts. In general, tumors with high TMB generally exhibited higher responses to immune checkpoint inhibition, which provided the basis for our follow-up study exploring the rate of CXCR4 predicted immune response in independent immunization cohorts [43]. In addition, IPS is calculated based on the gene expression of various factors such as immune effector cells, suppressor cells, and immune modulators in tumor samples [44, 45]. Although no significant difference in IPS was observed in LUAD, IPS was significantly increased in the CXCR4 high expression group in LUSC, reflecting the potential for better immunotherapy responsiveness in LUSC compared to LUAD, which was also confirmed in our follow-up study. More importantly, we included more accurate immunotherapy predictors (TIDE algorithm) to further demonstrate the immune effector potential of CXCR4. As expected, NSCLC patients with high CXCR4 expression had lower TIDE scores. Studies have shown that low TIDE scores are associated with better immunotherapy outcomes [27].

Considering the potential of CXCR4 in modulating the TME, we analyzed its role in immunotherapy response in an independent cohort. Among 13 LUAD patients who received immunotherapy, four showed PR, three of which also showed elevated CXCR4 expression. Moreover, the anti-PD-1 immunotherapy response rate was higher in the high CXCR4 expression group. Among 33 LUSC patients who received immunotherapy, two achieved CR status, 20 achieved PR status, and 11 patients were stable disease (SD) status. High CXCR4 expression was consistent with better treatment status in LUSC patients and was significantly associated with immunotherapy response. ROC curves revealed that CXCR4 showed excellent performance in predicting the efficacy of immunotherapy in both the LUAD and LUSC cohorts. Furthermore, our further IHC staining of 26 samples from the immunotherapy cohort found no significant difference between CXCR4 expression at the protein level and immunotherapy response. The reason may be that the sample size is too small and the protein expression is affected after treatment, which needs to be further verified by expanding the sample. Recent studies have shown that CXCR4 inhibitors can enhance T cell and B cell infiltration and induce immune responses in pancreatic and colorectal cancers [46]. Another study showed that PD-1 blockade combined with CXCR4 inhibition and sorafenib inhibited hepatocellular carcinoma growth [47]. Therefore, CXCR4 may be valuable in predicting immunotherapy in NSCLC patients. Moreover, we performed tumor tissue- and cell-level GSEA to determine the possible molecular mechanisms by which CXCR4 manifests its effects in NSCLC and found that MYC targets, DNA repair, and inflammation are associated with CXCR4 expression in LUAD, while the Notch signaling pathway and epithelial–mesenchymal transition are associated with CXCR4 expression in LUSC. The Notch signaling pathway has been shown to be involved in the occurrence and development of a variety of cancers; it is highly active and mediates apoptosis in LUSC, and inhibits tumor growth in LUAD [48, 49]. Moreover, glycosaminoglycan degradation, long-term depression, phosphatidylinositol signaling system, and galactose metabolism were the most enriched signaling pathways in the RNA-seq data of 118 lung cancer cell lines. Together, these findings suggest that CXCR4 can significantly regulate the biological pathways and the immune TME of NSCLC.

Previous studies have shown that CXCR4 is highly expressed in NSCLC and is related to the prognosis of patients [21]; however, the same in both the LUAD and LUSC subgroups has not been shown before. We confirmed the prognostic value of CXCR4 in LUAD and LUSC patients based on an independent cohort—high CXCR4 expression predicts poor patient prognosis. This phenomenon can be explained by the above findings that the high expression of CXCR4 is accompanied by the high expression of immune checkpoints, and patients are often inhibited in the body's anti-tumor immune activity, resulting in poor prognosis. At the same time, the advent of immune checkpoint inhibitors could change this situation, making patients with high CXCR4 expression more likely to benefit from immunotherapy. It should be noted that in the online survival analysis website Kaplan–Meier plotter, the prognostic results of CXCR4 were completely opposite to those in this study. The KM plotter database is a commonly used survival analysis tool for researchers, which aggregates transcriptomic and clinical data from multiple databases, such as TCGA, Gene Expression Omnibus (GEO), and European Genome-phenome Archive (EGA) [50]. The reason for the opposite results of CXCR4 in the KM plotter was mainly due to the inconsistency between the protein levels of the independent cohorts and the RNA levels of the public data. In addition, platform differences can also lead to differences in RNA and protein expression.

In addition, we identified CXCR4-related immunomodulators—19 immunostimulators and 11 immunoinhibitors—which were shown to participate in biological pathways such as lymphocyte activation, leukocyte cell–cell adhesion, cytokine-cytokine receptor interaction, and cell adhesion molecules by GO and KEGG enrichment analysis. Importantly, we constructed an immune prognostic signature based on 12 genes and the CXCR4-related immunomodulators HAVCR2, TGFBR1, CD40, CD40LG, CD70, CD80, CXCR4, ENTPD1, KLRK1, MICB, TNFRSF14, and TNFRSF17 in LUAD patients, and calculated the risk score of each subject to establish a model that can predict patient prognosis. HAVCR2 (Tim-3) has been shown to be expressed in the TME of NSCLC and affect T cell activation and can be used as a potential modulator of cancer immunotherapy to enhance the anti-tumor effect of checkpoint inhibitors [51, 52]. TGFBR1 inhibitors can significantly inhibit the growth of tumor cells in lung cancer lacking the tumor suppressor GARA4 and can be used as a potential therapeutic target [53]. Furthermore, we constructed an immune prognostic signature based on 16 genes and CXCR4-related immunomodulators, namely ADORA2A, BTLA, CTLA4, IDO1, TGFBR1, C10orf54, CD70, HHLA2, ICOSLG, IL6, IL6R, KLRC1, KLRK1, PVR, TNFRSF13C and TNFRSF18 in LUSC patients to establish a prognostic model. BTLA is highly expressed in tumor cells of NSCLC patients, which is significantly positively correlated with high levels of PD-L1 and can predict the poor prognosis of NSCLC patients [54]. Several expression models based on CD70 in breast cancer are used as biomarkers of lung-specific metastasis [55]. HHLA2 is significantly related to EGFR mutations and tumor-infiltrating lymphocyte density in LUAD patients. It is likely to become a new target for cancer immunotherapy, but its expression in LUSC needs to be further explored [56]. Elevated levels of PD-L1 and IDO1 in NSCLC patients have been shown to be associated with the infiltration of B and T cells, and potentially affect the immune escape of lung cancer cells [57]. Furthermore, other genes in the immune prognostic signature also play important roles in various cancers. The good predictive power of the constructed signatures was confirmed by the ROC curve. Univariate and multivariate Cox regression suggested that risk score is an independent prognostic factor for LUAD patients, and age and risk score as independent prognostic factors for LUSC patients. To further evaluate the efficacy of generating signatures based on immunomodulators to predict prognosis, we constructed a nomogram to further highlight the good predictive value of risk scores for each NSCLC patient. For the advantages of the prognostic signature in this study, we found a study on the development of immune-related signatures based on the Immunology Database and Analysis Portal (ImmPort) database. The ImmPort database was established in 2014 [58], and its transcriptome data and immune-related information are not as rich as the TISIDB database we used (the TISIDB database was published in 2019), and the signature predicts 3-year and 5-year survival AUC values in the range of 0.6–0.7. In addition, we also found a signature that was also constructed based on the TISIDB database, and the predictive power of the signature was 0.778 and 0.698 in LUAD and LUSC, respectively, and there was no more detailed study of key immune genes [59]. In the present study, we first demonstrated the ability of CXCR4 to predict prognosis and immunotherapy response in NSCLC patients using an IHC cohort and an immunotherapy cohort. Given the excellent performance of CXCR4, we recruited the immune genes most associated with CXCR4 based on the TISIDB database to construct a prognostic signature whose best AUCs were 0.816 and 0.817 in LUAD and LUSC, respectively, indicating better predictive power. At the same time, we believe that this study can provide researchers with a novel idea.

Our study is limited in that it was a single-center study, and multi-center large-scale samples are needed to verify the value of CXCR4 in the diagnosis and treatment of NSCLC. Furthermore, the immune prognostic signatures we constructed are based on public data, and their reliability should be verified in independent groups. The multiple CXCR4-related signaling pathways identified by GSEA should also be validated in in vivo and in vitro experiments, which will be addressed in our future studies.

In summary, our study initially showed the importance of the immune TME in NSCLC patients and determined the correlation between CXCR4 and NSCLC immune cell infiltration in NSCLC. We demonstrated the powerful and reliable prognostic value and performance of CXCR4 in predicting immunotherapy response in two NSCLC independent cohorts. More importantly, the prognostic signatures of CXCR4-related immunomodulators constructed were shown accurately predict the survival status of LUAD and LUSC patients and should be implemented in clinical settings.

### Supplementary Information

Below is the link to the electronic supplementary material.

Supplementary file1 (TIF 47989 KB)

Supplementary file2 (TIF 10835 KB)

Supplementary file3 (TIF 4001 KB)

Supplementary file4 (TIF 16156 KB)

Supplementary file5 (TIF 7951 KB)

Supplementary file6 (TIF 8060 KB)

Supplementary file7 (TIF 4488 KB)

Supplementary file8 (TIF 4385 KB)

Supplementary file9 (JPG 1168 KB)

Supplementary file10 (TIF 6538 KB)

Supplementary file11 (TIF 1927 KB)

Supplementary file12 (XLSX 12 KB)

Supplementary file13 (DOCX 17 KB)

Supplementary file14 (DOCX 17 KB)

Supplementary file15 (DOCX 19 KB)

Supplementary file16 (DOCX 19 KB)

Supplementary file17 (DOCX 19 KB)

## Data Availability

All data generated or analyzed based on public databases in this study were obtained from the TCGA, TIMER, TCIA, CCLE and TISIDB databases, and relevant descriptions and website links are included in this published article. For independent cohort experimental data can be obtained from the corresponding author upon reasonable request.
