# Supplementary figures and images for: Comprehensive analysis of the immunological implication and prognostic value of CXCR4 in non-small cell lung cancer
Source: Cancer Immunol Immunother. 2022 Oct 29;72(4):1029–45. doi: 10.1007/s00262-022-03298-y (PMC10025233; doi:10.1007/s00262-022-03298-y)

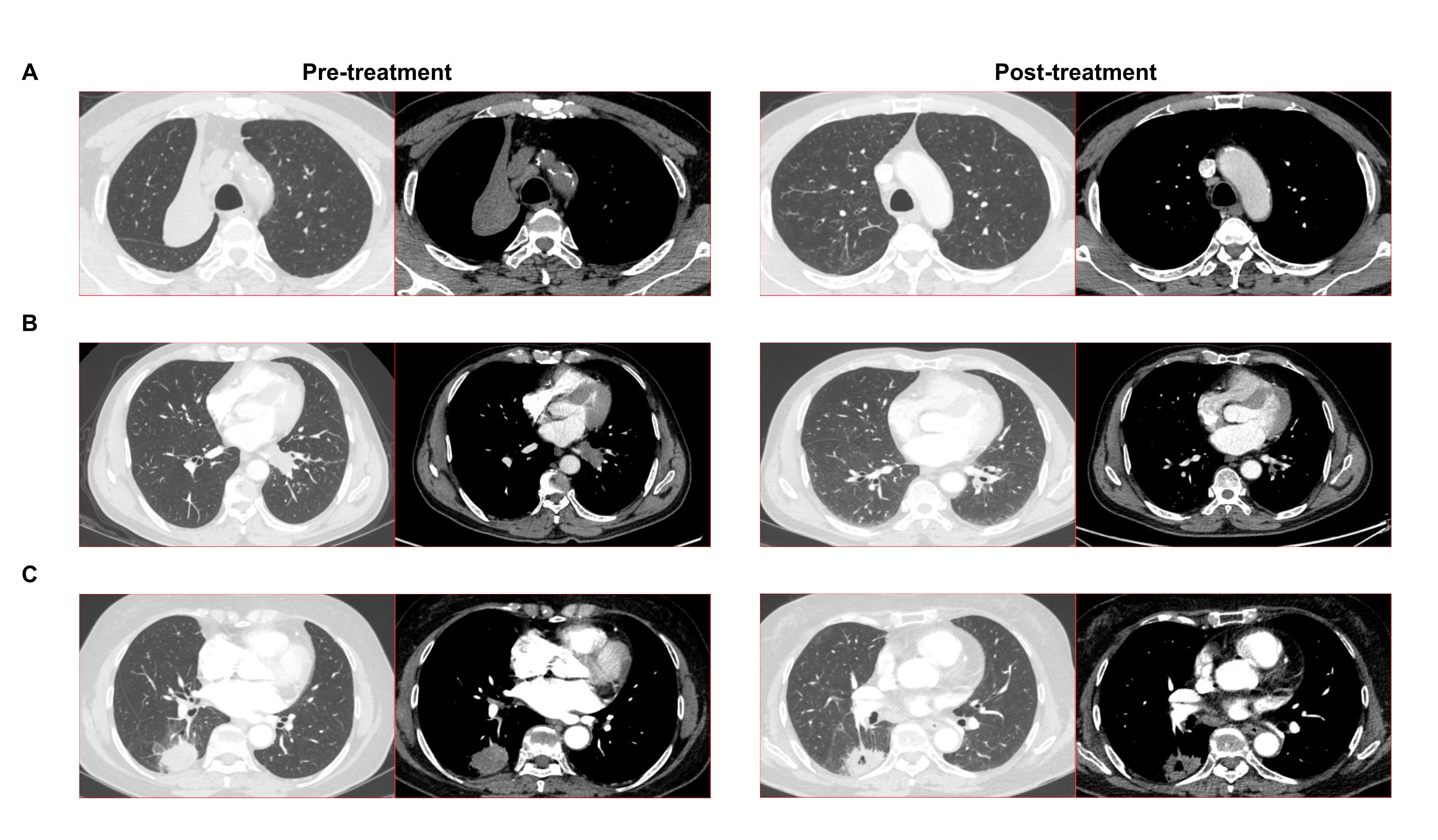

Supplement: Supplementary file 1 — Supplementary file1 (TIF 47989 KB) [file 262_2022_3298_MOESM1_ESM.tif]

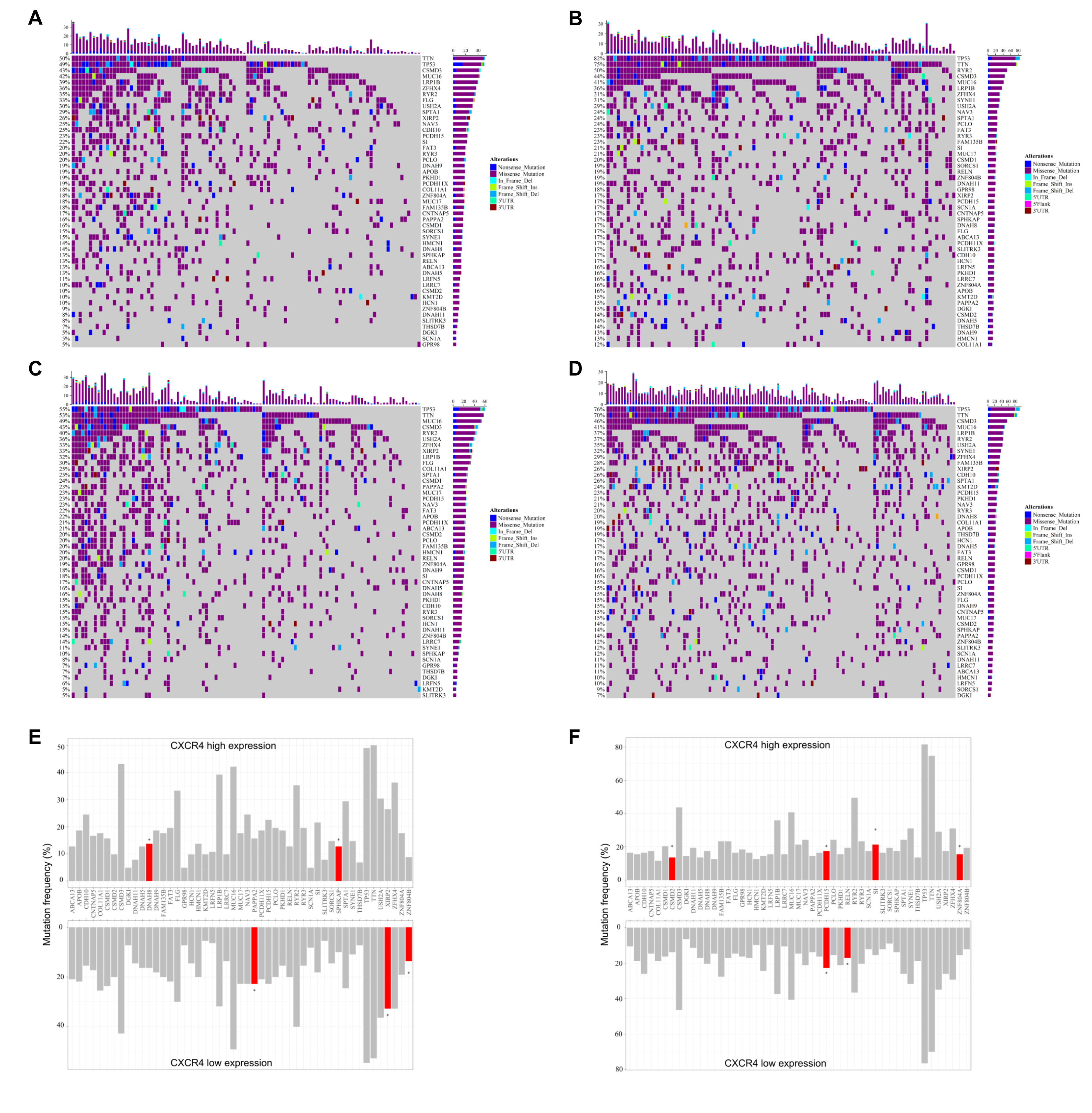

Supplement: Supplementary file 2 — Supplementary file2 (TIF 10835 KB) [file 262_2022_3298_MOESM2_ESM.tif]

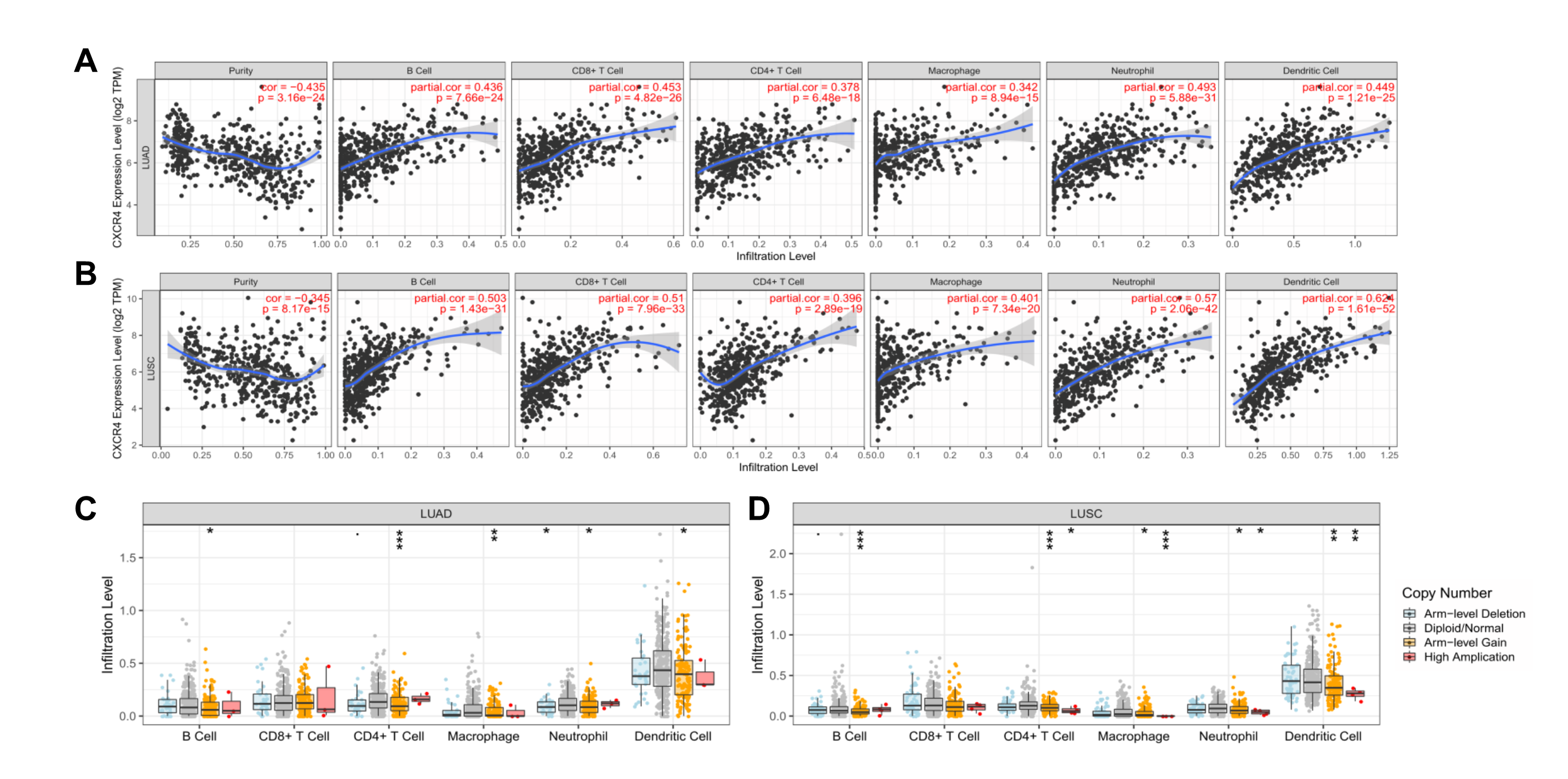

Supplement: Supplementary file 3 — Supplementary file3 (TIF 4001 KB) [file 262_2022_3298_MOESM3_ESM.tif]

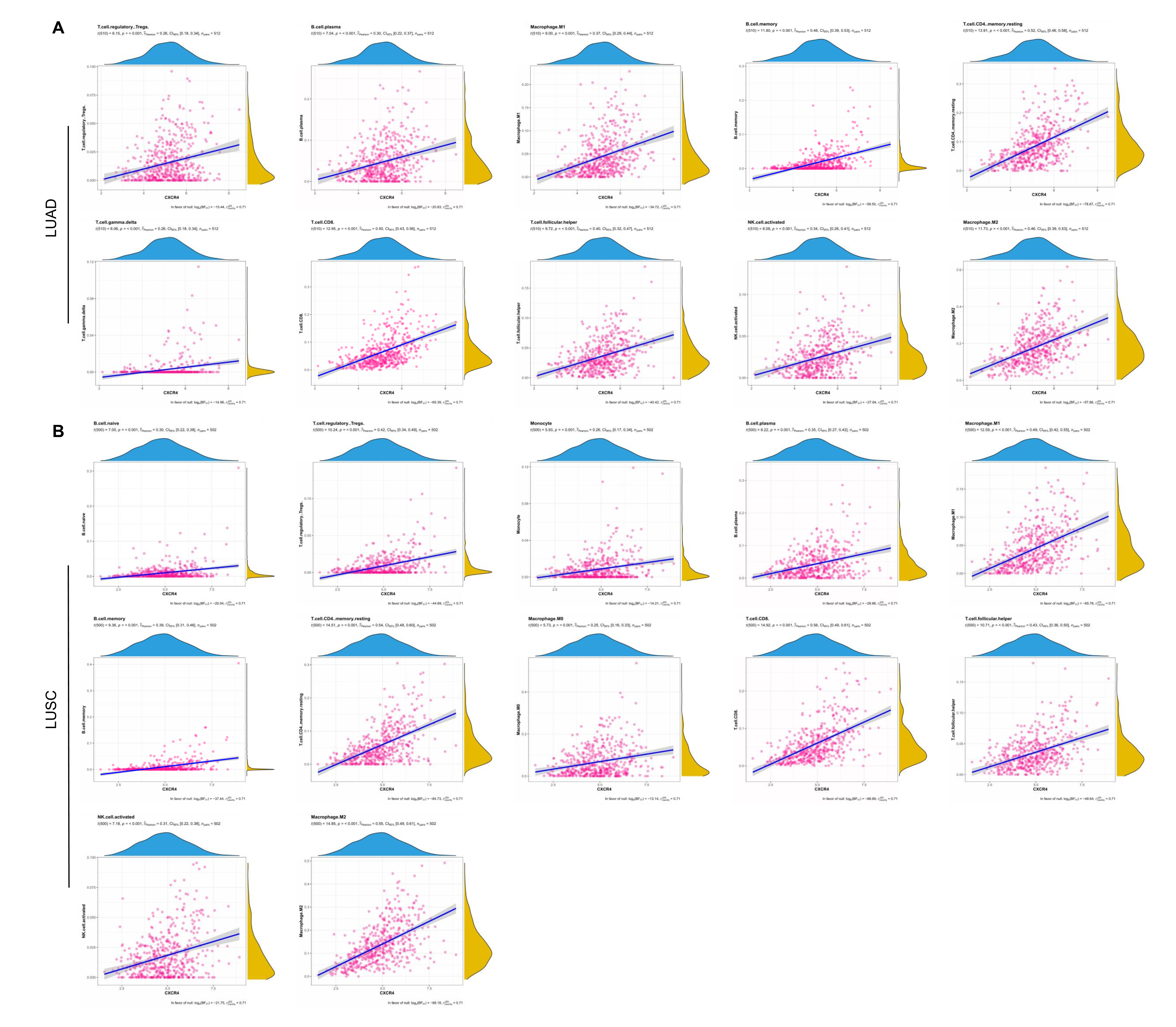

Supplement: Supplementary file 4 — Supplementary file4 (TIF 16156 KB) [file 262_2022_3298_MOESM4_ESM.tif]

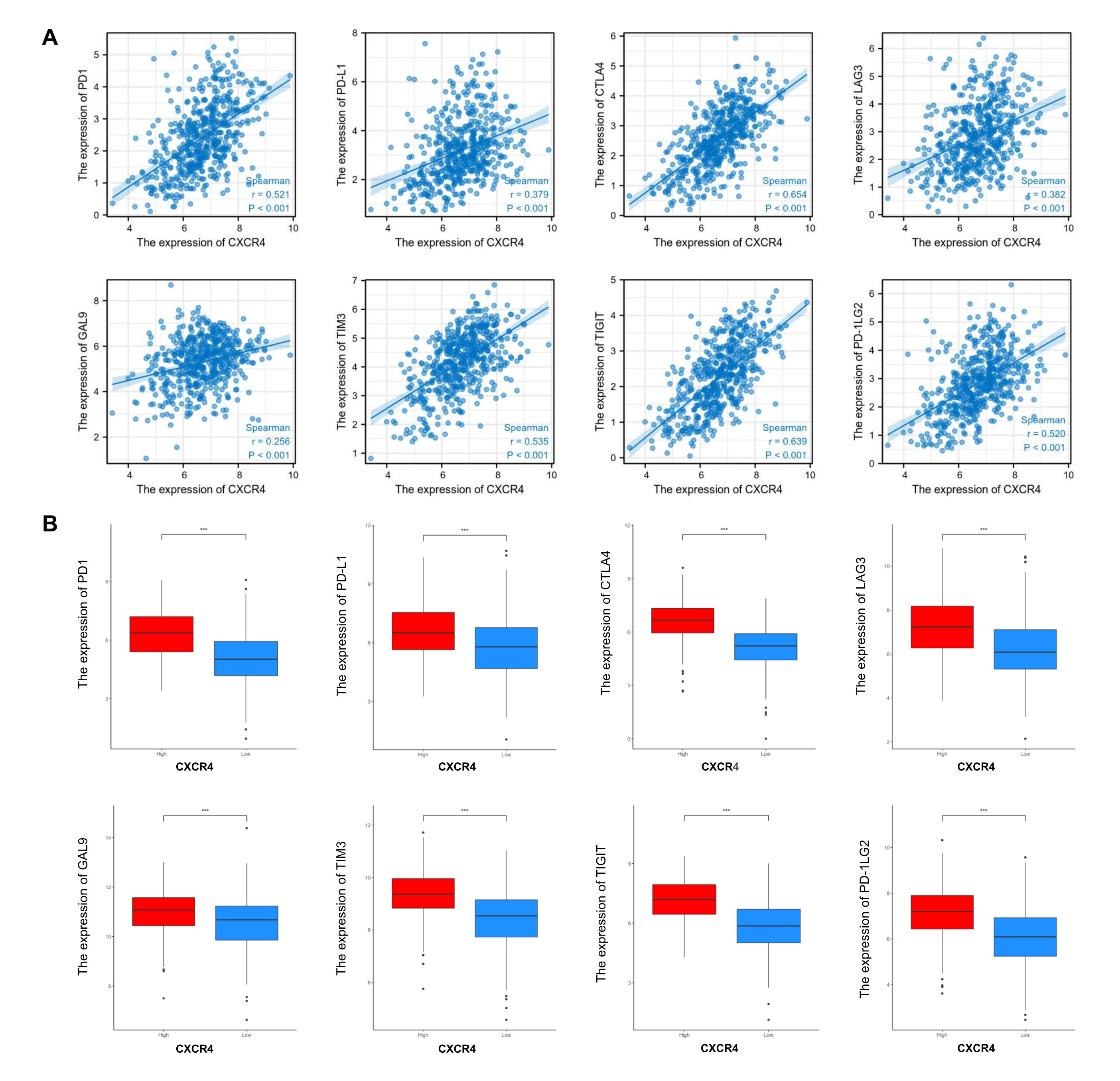

Supplement: Supplementary file 5 — Supplementary file5 (TIF 7951 KB) [file 262_2022_3298_MOESM5_ESM.tif]

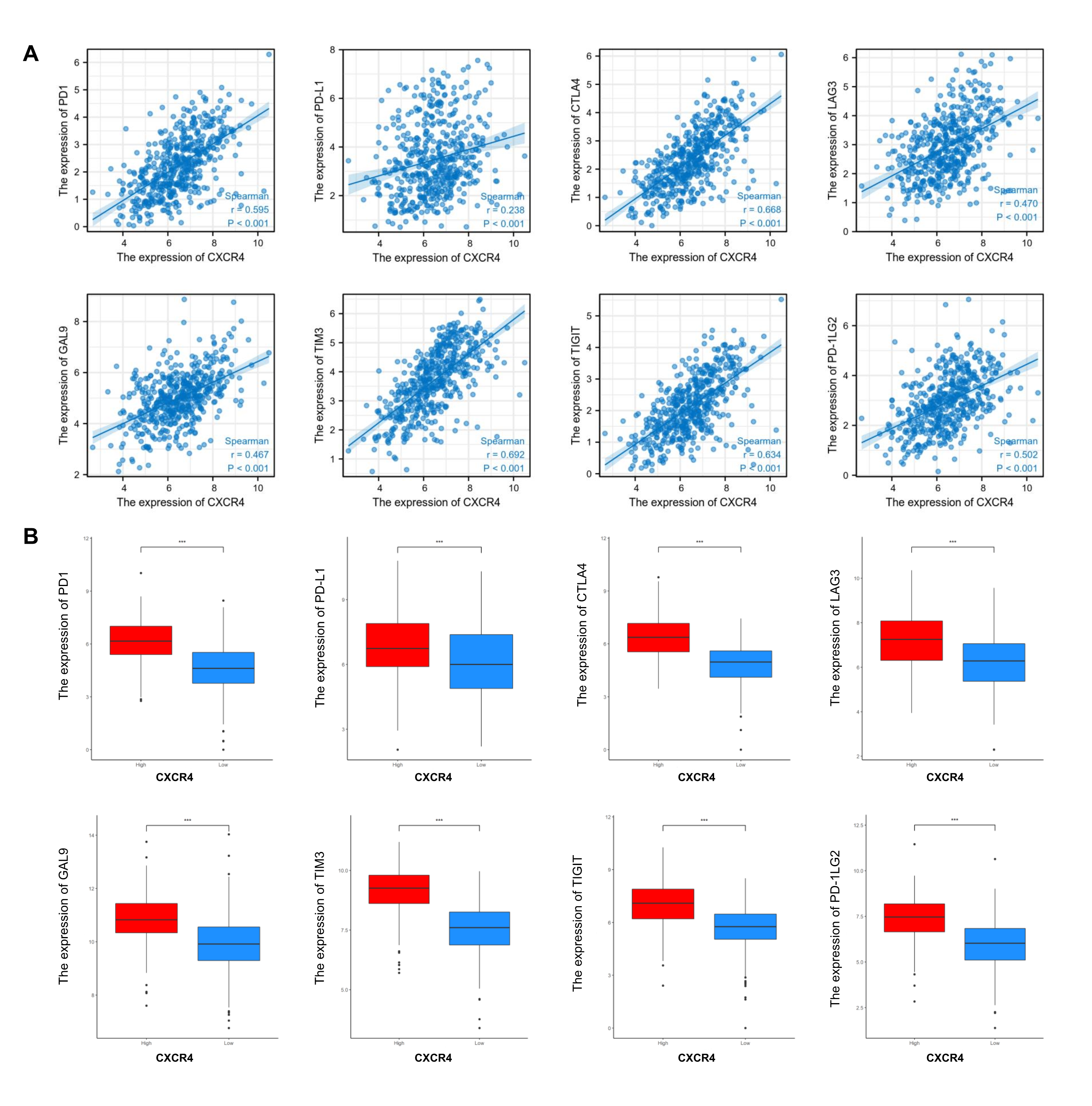

Supplement: Supplementary file 6 — Supplementary file6 (TIF 8060 KB) [file 262_2022_3298_MOESM6_ESM.tif]

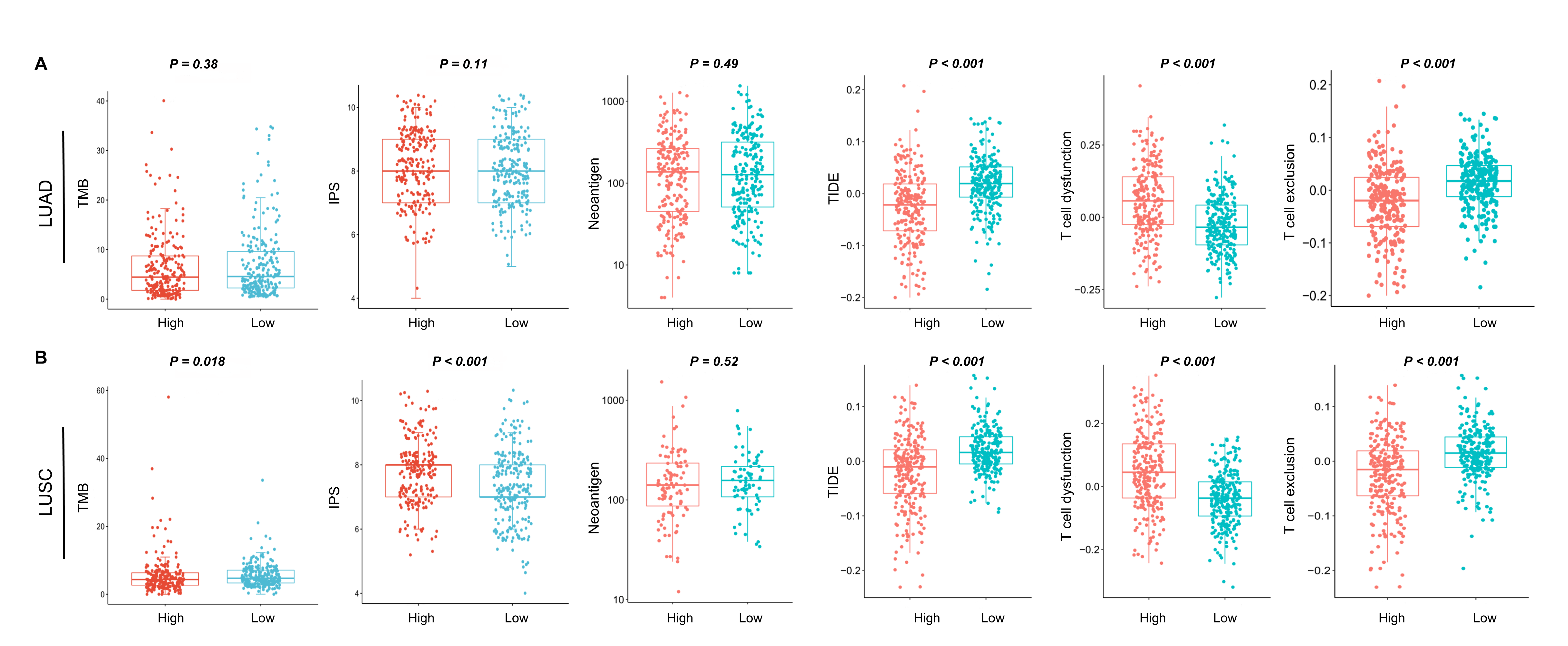

Supplement: Supplementary file 7 — Supplementary file7 (TIF 4488 KB) [file 262_2022_3298_MOESM7_ESM.tif]

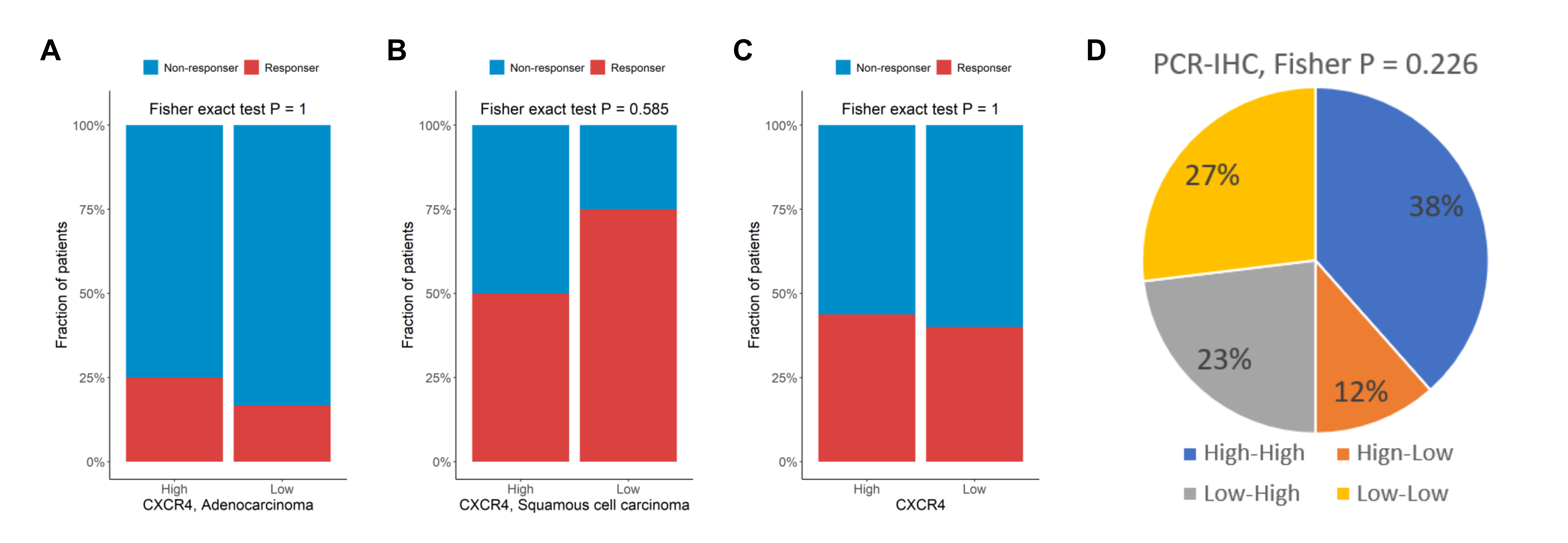

Supplement: Supplementary file 8 — Supplementary file8 (TIF 4385 KB) [file 262_2022_3298_MOESM8_ESM.tif]

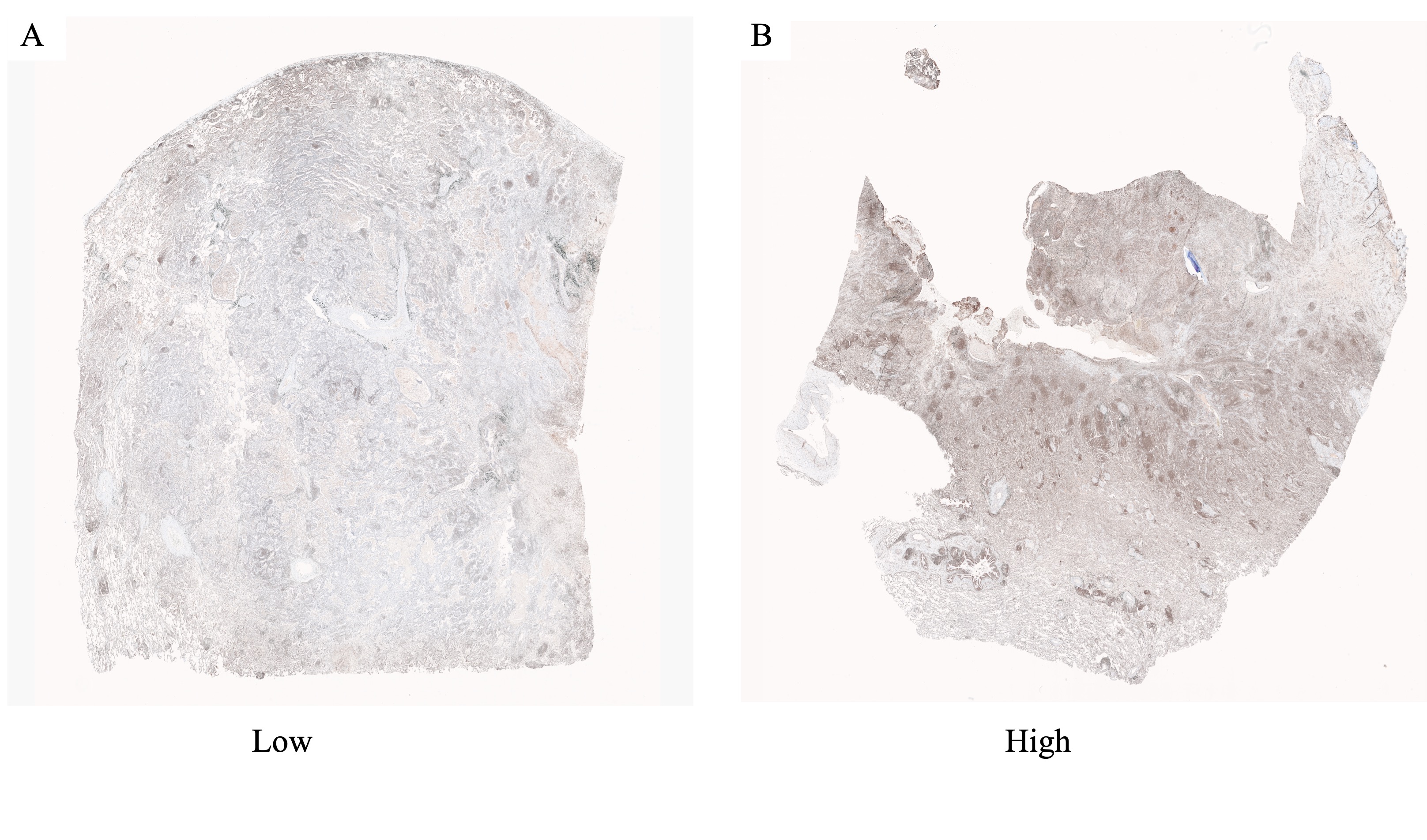

Supplement: Supplementary file 9 — Supplementary file9 (JPG 1168 KB) [file 262_2022_3298_MOESM9_ESM.jpg]

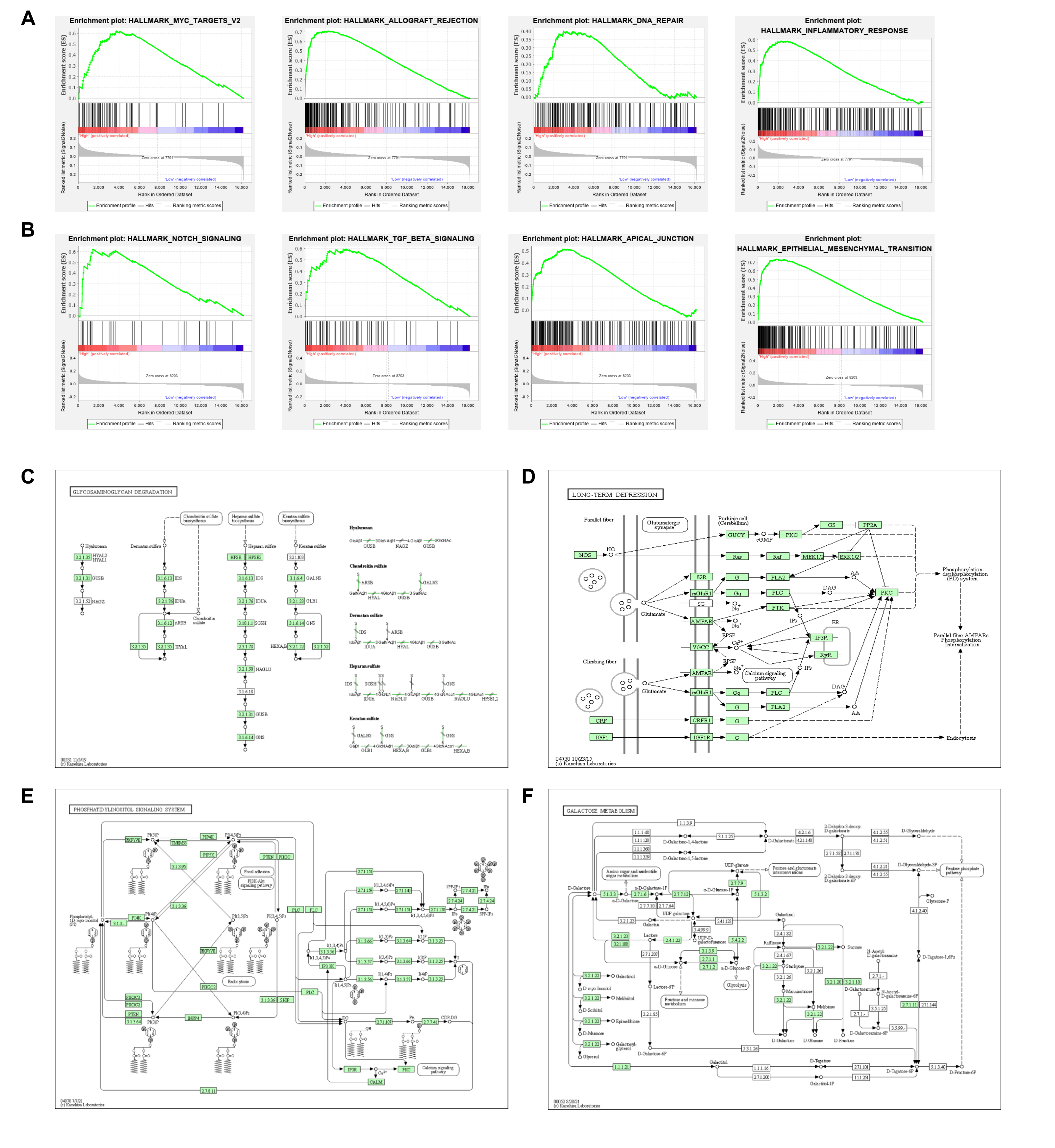

Supplement: Supplementary file 10 — Supplementary file10 (TIF 6538 KB) [file 262_2022_3298_MOESM10_ESM.tif]

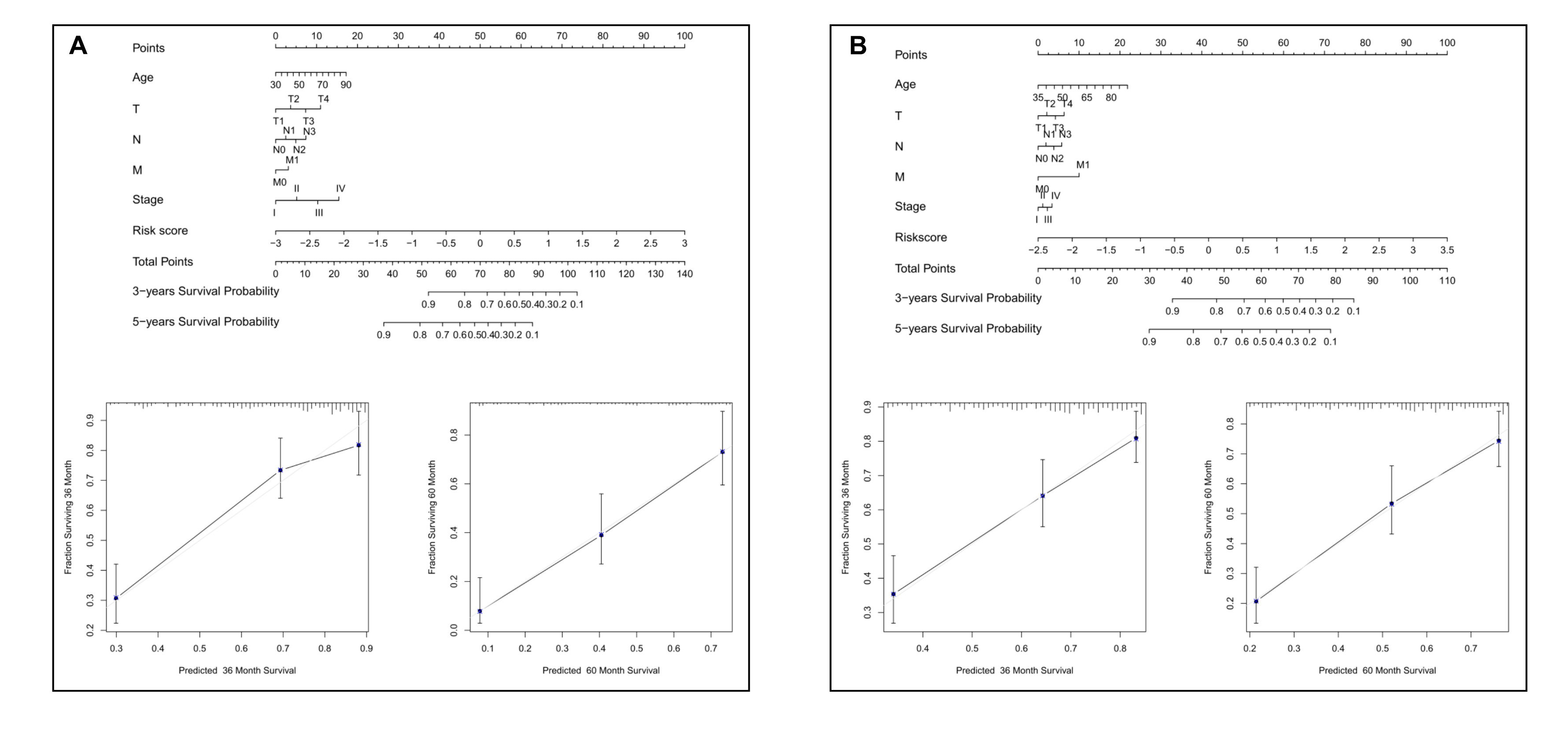

Supplement: Supplementary file 11 — Supplementary file11 (TIF 1927 KB) [file 262_2022_3298_MOESM11_ESM.tif]
