# Supplementary material for: Comprehensive analysis of the immunological implication and prognostic value of CXCR4 in non-small cell lung cancer
Source: Cancer Immunol Immunother. 2022 Oct 29;72(4):1029–45. doi: 10.1007/s00262-022-03298-y (PMC10025233; doi:10.1007/s00262-022-03298-y)
Supplement: Supplementary file 13 — Supplementary file13 (DOCX 17 KB) [file 262_2022_3298_MOESM13_ESM.docx]

| Rank | Name of pathway | ES | NES | NOM p-value | FDR q-value | FWER p-value |
| --- | --- | --- | --- | --- | --- | --- |
| 1 | MYC_TARGETS_V2 | 0.62 | 1.93 | 0.005 | 0.017 | 0.017 |
| 2 | ALLOGRAFT_REJECTION | 0.56 | 1.87 | 0.009 | 0.015 | 0.028 |
| 3 | DNA_REPAIR | 0.40 | 1.70 | 0.019 | 0.068 | 0.159 |
| 4 | INFLAMMATORY_RESPONSE | 0.58 | 1.56 | 0.042 | 0.135 | 0.424 |
| 5 | IL2_STAT5_SIGNALING | 0.49 | 1.54 | 0.025 | 0.132 | 0.453 |
| 6 | IL6_JAK_STAT3_SIGNALING | 0.58 | 1.51 | 0.046 | 0.140 | 0.502 |
| 7 | MTORC1_SIGNALING | 0.44 | 1.50 | 0.058 | 0.135 | 0.523 |
| 8 | COMPLEMENT | 0.47 | 1.49 | 0.057 | 0.131 | 0.543 |
| 9 | PI3K_AKT_MTOR_SIGNALING | 0.35 | 1.44 | 0.072 | 0.160 | 0.624 |
| 10 | INTERFERON_GAMMA_RESPONSE | 0.55 | 1.43 | 0.121 | 0.154 | 0.639 |

Table S1. Top 10 Biological Processes Enriched in LUAD Based on CXCR4

ES, enrichment score; NES, normalized enrichment score; NOM, nominal p-value; FDR, false discovery rate; FWER, familywise-error rate; LUAD, Lung adenocarcinoma.
