# Supplementary material for: Comprehensive analysis of the immunological implication and prognostic value of CXCR4 in non-small cell lung cancer
Source: Cancer Immunol Immunother. 2022 Oct 29;72(4):1029–45. doi: 10.1007/s00262-022-03298-y (PMC10025233; doi:10.1007/s00262-022-03298-y)
Supplement: Supplementary file 14 — Supplementary file14 (DOCX 17 KB) [file 262_2022_3298_MOESM14_ESM.docx]

| Rank | Name of pathway | ES | NES | NOM p-value | FDR q-value | FWER p-value |
| --- | --- | --- | --- | --- | --- | --- |
| 1 | NOTCH_SIGNALING | 0.62 | 1.65 | 0.018 | 0.243 | 0.222 |
| 2 | TGF_BETA_SIGNALING | 0.59 | 1.60 | 0.024 | 0.245 | 0.308 |
| 3 | APICAL_JUNCTION | 0.51 | 1.59 | 0.007 | 0.203 | 0.326 |
| 4 | EPITHELIAL_MESENCHYMAL_TRANSITION | 0.73 | 1.59 | 0.012 | 0.163 | 0.328 |
| 5 | HEDGEHOG_SIGNALING | 0.60 | 1.56 | 0.027 | 0.165 | 0.371 |
| 6 | APICAL_SURFACE | 0.55 | 1.51 | 0.014 | 0.218 | 0.486 |
| 7 | PROTEIN_SECRETION | 0.34 | 1.48 | 0.073 | 0.229 | 0.547 |
| 8 | MYOGENSIS | 0.50 | 1.46 | 0.029 | 0.224 | 0.567 |
| 9 | ANGIOGENESIS | 0.70 | 1.45 | 0.054 | 0.213 | 0.583 |
| 10 | INFLAMMATORY_RESPONSE | 0.52 | 1.36 | 0.119 | 0.347 | 0.748 |

Table S2. Top 10 Biological Processes Enriched in LUSC Based on CXCR4

ES, enrichment score; NES, normalized enrichment score; NOM, nominal p-value; FDR, false discovery rate; FWER, familywise-error rate; LUSC, Lung squamous cell carcinoma.
