# Supplementary material for: Comprehensive analysis of the immunological implication and prognostic value of CXCR4 in non-small cell lung cancer
Source: Cancer Immunol Immunother. 2022 Oct 29;72(4):1029–45. doi: 10.1007/s00262-022-03298-y (PMC10025233; doi:10.1007/s00262-022-03298-y)
Supplement: Supplementary file 15 — Supplementary file15 (DOCX 19 KB) [file 262_2022_3298_MOESM15_ESM.docx]

| Rank | Name of pathway | ES | NES | NOM p-value | FDR q-value | FWER p-value |
| --- | --- | --- | --- | --- | --- | --- |
| 1 | GLYCOSAMINOGLYCAN_DEGRADATION | 0.48 | 1.77 | 0.004 | 0.497 | 0.262 |
| 2 | LONG_TERM_DEPRESSION | 0.43 | 1.75 | 0.002 | 0.282 | 0.291 |
| 3 | PHOSPHATIDYLINOSITOL_SIGNALING | 0.49 | 1.68 | 0.004 | 0.374 | 0.459 |
| 4 | GALACTOSE_METABOLISM | 0.49 | 1.67 | 0.026 | 0.294 | 0.471 |
| 5 | NON_SMALL_CELL_LUNG_CANCER | 0.45 | 1.65 | 0.008 | 0.277 | 0.510 |
| 6 | BASAL_TRANSCRIPTION_FACTORS | 0.53 | 1.64 | 0.022 | 0.210 | 0.527 |
| 7 | DNA_REPLICATION | 0.63 | 1.63 | 0.046 | 0.203 | 0.551 |
| 8 | LYSINE_DEGRADATION | 0.48 | 1.59 | 0.023 | 0.238 | 0.643 |
| 9 | LONG_TERM_POTENTIATION | 0.44 | 1.59 | 0.017 | 0.218 | 0.651 |
| 10 | ERBB_SIGNALING_PATHWAY | 0.40 | 1.58 | 0.006 | 0.210 | 0.670 |

Table S3. Top 10 KEGG pathways enriched in 118 lung cancer cell lines from CCLE based on CXCR4

ES, enrichment score; NES, normalized enrichment score; NOM, nominal p-value; FDR, false discovery rate; FWER, familywise-error rate.
