# Supplementary material for: Comprehensive analysis of the immunological implication and prognostic value of CXCR4 in non-small cell lung cancer
Source: Cancer Immunol Immunother. 2022 Oct 29;72(4):1029–45. doi: 10.1007/s00262-022-03298-y (PMC10025233; doi:10.1007/s00262-022-03298-y)
Supplement: Supplementary file 16 — Supplementary file16 (DOCX 19 KB) [file 262_2022_3298_MOESM16_ESM.docx]

Table 3. Univariate and multivariable analysis of factors associated with overall survival in NCC LUAD cohort

|  | Univariate analysis | | | Multivariate analysis | | |
| --- | --- | --- | --- | --- | --- | --- |
|  | P value | HR | 95%CI | P value | HR | 95%CI |
| Age  (≤60, >60years) | **<0.001** | 1.844 | 1.345-2.529 | **<0.001** | 1.980 | 1.417-2.766 |
| Gender  (female, male) | 0.207 | 0.814 | 0.592-1.120 |  |  |  |
| Smoking  (never, ever) | 0.112 | 1.286 | 0.943-1.754 |  |  |  |
| Tumor length (cm)  ≤4  >4 | **<0.001** | 2.154 | 1.575-2.947 | **0.002** | 1.740 | 1.234-2.452 |
| Differentiation  (well/moderate, poor) | **0.005** | 1.574 | 1.143-2.167 | **0.035** | 1.437 | 1.025-2.015 |
| T stage  (T1-T2, T3-T4) | **0.039** | 1.462 | 1.019-2.099 | 0.524 | 1.144 | 0.756-1.730 |
| lymph node metastasis  (negative, positive) | **<0.001** | 2.276 | 1.618-3.202 | **0.033** | 1.651 | 1.040-2.621 |
| TNM stage  (I/II, III) | **<0.001** | 1.919 | 1.405-2.620 | 0.896 | 1.029 | 0.667-1.569 |
| CXCR4 expression  (negative, positive) | **<0.001** | 3.184 | 2.303-4.404 | **<0.001** | 2.988 | 2.138-4.175 |

LUAD, lung adenocarcinoma
