# Supplementary material for: Comprehensive analysis of the immunological implication and prognostic value of CXCR4 in non-small cell lung cancer
Source: Cancer Immunol Immunother. 2022 Oct 29;72(4):1029–45. doi: 10.1007/s00262-022-03298-y (PMC10025233; doi:10.1007/s00262-022-03298-y)
Supplement: Supplementary file 17 — Supplementary file17 (DOCX 19 KB) [file 262_2022_3298_MOESM17_ESM.docx]

Table 4. Univariate and multivariable analysis of factors associated with overall survival in NCC LUSC cohort

|  | Univariate analysis | | | Multivariate analysis | | |
| --- | --- | --- | --- | --- | --- | --- |
|  | P value | HR | 95%CI | P value | HR | 95%CI |
| Age  (≤60, >60years) | 0.113 | 1.361 | 0.930-1.991 |  |  |  |
| Gender  (female, male) | 0.551 | 1.418 | 0.450-4.469 |  |  |  |
| Smoking  (never, ever) | 0.970 | 1.015 | 0.472-2.182 |  |  |  |
| Tumor length (cm)  ≤4  >4 | **0.018** | 1.597 | 1.082-2.357 | 0.617 | 0.878 | 0.526-1.464 |
| Differentiation  (well/moderate, poor) | **0.037** | 1.499 | 1.025-2.191 | **0.050** | 1.466 | 1-2.148 |
| T stage  (T1-T2, T3-T4) | **<0.001** | 2.734 | 1.874-4.015 | **0.033** | 1.901 | 1.053-3.431 |
| Lymph node metastasis  (negative, positive) | **0.001** | 2.084 | 1.341-3.239 | 0.425 | 1.255 | 0.718-2.192 |
| TNM stage  (I-II, III) | **<0.001** | 2.824 | 1.922-4.148 | **0.044** | 1.834 | 1.017-3.307 |
| CXCR4 expression  (negative, positive) | 0.064 | 1.429 | 0.979-2.085 | 0.062 | 1.448 | 0.982-2.135 |

LUSC, lung squamous cell carcinoma.
